# Boranediyl‐ and Diborane(4)‐1,2‐diyl‐Bridged Platinum A‐Frame Complexes

**DOI:** 10.1002/chem.202001168

**Published:** 2020-06-17

**Authors:** Carina Brunecker, Jonas H. Müssig, Merle Arrowsmith, Felipe Fantuzzi, Andreas Stoy, Julian Böhnke, Alexander Hofmann, Rüdiger Bertermann, Bernd Engels, Holger Braunschweig

**Affiliations:** ^1^ Institut für Anorganische Chemie and Institute for Sustainable Chemistry & Catalysis with Boron, Julius-Maximilians-Universität Wüzburg Am Hubland 97074 Würzburg Germany; ^2^ Institut für Physikalische und Theoretische Chemie Julius-Maximilians-Universität Würzburg Emil-Fischer-Straße 42 97074 Würzburg Germany

**Keywords:** boron, bonding, EDA-NOCV, oxidative addition, platinum

## Abstract

Diplatinum A‐frame complexes with a bridging (di)boron unit in the apex position were synthesized in a single step by the double oxidative addition of dihalo(di)borane precursors at a bis(diphosphine)‐bridged Pt^0^_2_ complex. While structurally analogous to well‐known *μ‐*borylene complexes, in which delocalized dative three‐center‐two‐electron M‐B‐M bonding prevails, theoretical investigations into the nature of Pt−B bonding in these A‐frame complexes show them to be rare dimetalla(di)boranes displaying two electron‐sharing Pt−B σ‐bonds. This is experimentally reflected in the low kinetic stability of these compounds, which are prone to loss of the (di)boron bridgehead unit.

## Graphical abstract

**(Di)boron‐bridged platinum A‐frame complexes** were synthesized by the twofold oxidative addition of dihalo(di)boranes to a bis(diphosphine)‐bridged diplatinum(0) precursor. Experimental and theoretical studies show that these compounds are rare (di)boranediyl complexes with electron‐sharing Pt−B bonds.

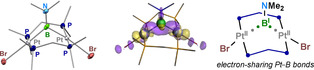

In organometallic chemistry, transition metal carbene complexes are divided into two classes: a) Fischer carbenes, in which R_2_C−M bonding is governed by σ donation of the carbene lone pair into an empty metal orbital and π backdonation from a filled metal d orbital into the empty carbene p orbital, and b) Schrock carbenes, in which bonding occurs between a triplet R_2_C: carbene and a triplet‐state metal center.^1^ With their singlet ground state, which is independent of the nature of the substituent R,^2^ and empty p orbitals, borylenes (RB:) may be considered as analogues of Fischer carbenes. Thus, the bonding in terminal borylene complexes is governed by σ donation from the borylene lone pair to the metal center and π backbonding from the electron‐rich metal center to the borylene, resulting in metal–boron multiple bonding.^3^

Like Schrock carbenes, however, borylenes are often found in bridging positions between two or more metal centers. Since the isolation of the first dinuclear bridging borylene complexes [*μ*‐(BY){*η* ^5^‐(C_5_H_4_R)Mn(CO)_2_}_2_] (Y=NMe_2_, R=H; Y=*t*Bu, R=Me),^4^ this class of compounds has been extensively studied in terms of electronic properties and reactivity.3a, 3b, 3c The nature of M‐B‐M bonding in borylene‐bridged dimanganese complexes (**I**, Figure 1) was examined both experimentally and computationally. Instead of the delocalized three‐center‐two‐electron (3c2e) dative bonding expected for bridging borylenes, the topological analysis of the electron density distribution from a low‐temperature, high‐resolution X‐ray diffraction experiment suggested two localized, directional two‐electron Mn−B bonds, leading the authors to describe these complexes as dimetallaboranes (or boranediyls).^5^ Calculations based on the quantum theory of atoms in molecules (QTAIM) and on the electron‐localization function (ELF) revealed that the calculated bonding situation—that is, borylene versus boranediyl—strongly depends on the choice of exchange‐correlation functional.^5^ Similar calculations on homodinuclear nickel (**II**) and cobalt (**III**) *μ*‐borylene complexes suggested that the dinickel complex should be described as a true bridging borylene whereas bonding in the dicobalt complex is closer to the boranediyl model, irrespective of the choice of density functional.^7^ Additionally, complexes **I**–**III** are all stabilized by delocalization of the metal–metal bonding molecular orbital over the empty orbitals of the boron bridge and thus fulfil the 18 valence electron rule.

**Figure 1:**
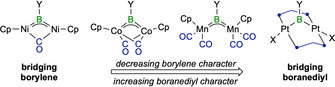
Bonding modes in homobimetallic *μ*‐BY‐bridged complexes. Cp = cyclopentadienyl.

Since the nature of the M‐B‐M bonding in bridging “borylene” complexes remains complicated to determine both experimentally and computationally, we set out to synthesize true boranediyl complexes and compare their structure, electronics and reactivity to known bridging borylenes. For this purpose, we chose so‐called A‐frame complexes,^8^ in which the two metal centers are tethered by two bridging diphosphine ligands and a bridging apex ligand, and which generally show no metal‐metal bonding interactions. In this communication we present the synthesis and characterization of a series of boranediyl platinum A‐frame complexes (**IV**), as well as a unique diborane(4)‐1,2‐diyl complex, and undertake an in‐depth computational analysis of the nature of bonding in the Pt‐B‐Pt bridge.

Our precursor for the desired boron‐bridged platinum A‐frames, complex **1**, was synthesized by reacting [Pt(nbe)_3_] (nbe=norbornene) with 2 equiv bis(dimethylphosphino)methane (dmpm) at 0 °C (Scheme 1). The ^31^P NMR spectrum of **1** shows a single resonance of higher order, similar to the literature‐known complex [(*μ*‐dppm)_3_Pt_2_] (dppm=bis(diphenylphosphino)methane).^9^ In the solid state the Pt⋅⋅⋅Pt distance of 4.096(1) Å confirms the absence of Pt−Pt bonding in **1**. To our knowledge, **1** is the only crystallographically characterized diphosphine‐bridged dinuclear Pt^0^ complex without Pt−Pt bonding.^10^

**Scheme 1:**
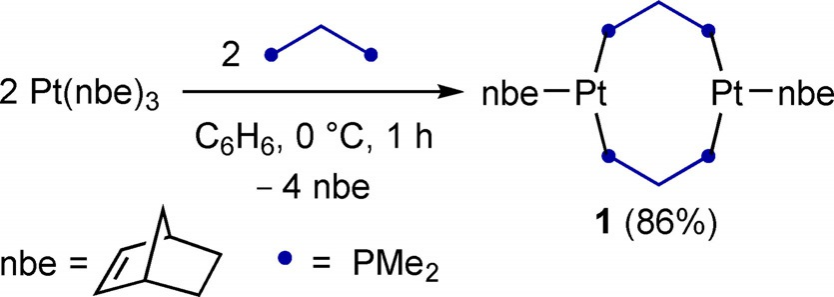
Synthesis of diplatinum complex **1**.

Previous syntheses of terminal platinum borylene complexes have generally relied on the oxidative addition of RBX_2_ dihaloboranes to Pt^0^ centers to generate boryl complexes of the form [L_2_PtX(BXR)], which were then converted to the corresponding cationic^11^ or neutral borylenes^12^ by halide abstraction or base‐induced halide transfer to platinum, respectively. With this in mind, we carried out the room‐temperature addition of dibromoboranes (BYBr_2_, Y=Dur=2,3,5,6‐Me_4_C_6_H, Br, NMe_2_) to a benzene solution of complex **1**, which led to the instant formation of a yellow–orange precipitate (Scheme 2 a). Recrystallization from a dichloromethane/pentane mixture at room temperature yielded the *μ*‐borylene diplatinum A‐frame complexes **2‐Dur** (66 %), **2‐Br** (34 %) and **2‐NMe_2_**, the latter proving highly unstable in both solution and the solid state (Scheme 3). By employing the dimethylsulfide precursor Me_2_S⋅BBr_3_ instead of BBr_3_ the yield of **2‐Br** became essentially quantitative (Scheme 2 b).

**Scheme 2:**
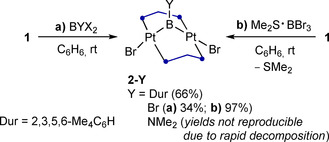
Synthesis of boranediyl‐bridged diplatinum A‐frame complexes.

**Scheme 3:**
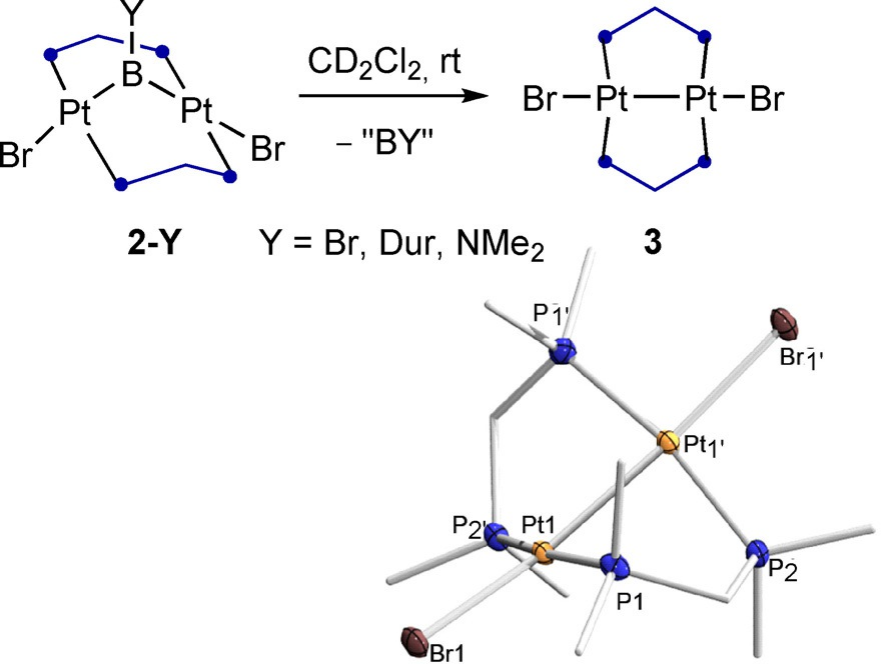
Decomposition of boranediyl complexes **2‐Y** to **3** and crystallographically derived molecular structure of **3**. Thermal ellipsoids at 50 % probability. Thermal ellipsoids of ligand periphery and hydrogen atoms omitted for clarity. Selected bond lengths (Å) and angles (°): Pt1−Pt1′ 2.6163(4), Pt1−Br1 2.5189(5), P1‐Pt1‐P2′ 169.53(4), Br1‐Pt1‐Pt1′ 172.859(11), torsion angle P1‐Pt1‐Pt1′‐P2 49.41(4).

The ^11^B NMR spectra of **2‐Y** show extremely broad resonances (full width at mid‐height (fwmh) ≈1200 to 2150 Hz) at 98 (**2‐Dur**), 85 (**2‐Br**) and 52 ppm (**2‐NMe_2_**), in line with the increasingly electron‐donating nature of the substituent at boron. These are significantly upfield‐shifted compared to structurally related borylene‐bridged bimetallic complexes (*μ*‐BAr: δ_11B_=122 to 162 ppm; *μ*‐BBr: δ_11B_=107 to 163 ppm; *μ*‐BNR_2_: δ_11B_=70 to 119 ppm).3b, 13 It is noteworthy, however, that the ^11^B NMR resonances of platinum‐containing heterobimetallic bridging borylenes, [*μ*‐BY{(ML_n_)(PtL_m_)}], are generally 20 to 35 ppm upfield‐shifted compared to the analogous homobimetallic complexes without platinum, [*μ*‐BY(ML_n_)_2_],^14^ owing to the strongly electron‐donating nature of the Pt^0^ center.

The ^31^P{^1^H} NMR spectra of **2‐Dur**, **2‐Br** and **2‐NMe_2_** showed singlets at −13.6, −9.3 and −5.6 ppm, respectively, with a complex higher‐order satellite motif. These arise from the superimposed spectra of isotopomers containing no ^195^Pt nuclei (43.8 %, sharp singlet), one ^195^Pt nucleus (44.8 %, AA′A′′A′′′X spin system), and two ^195^Pt nuclei (11.4 %, AA′A′′A′′′XX′ spin system), based on the natural abundance of these isotopes. Based on reported analyses of higher‐order spectra in doubly dppm‐bridged diplatinum complexes,^15^ the ^31^P{^1^H} NMR spectrum of **2‐Br** (Figure 2) yields coupling constants of ^1^ *J*_PPt_=3270 Hz, *J*_P1Pt2_=220 Hz, *J*_PtPt_=510 Hz and *Q=J*_P1P2_+*J*_P1P3_=42 Hz.^16^ These coupling constants are similar to those observed for cationic *μ*‐hydride^15^ and *μ*‐chloride‐bridged15b and neutral CH_2_‐bridged^17^ (*μ*‐dppm)_2_Pt_2_ A‐frame complexes. The ^195^Pt{^1^H} NMR spectrum of **2‐Br** displays a higher‐order multiplet at −3865 ppm, additionally broadened by coupling to the quadrupolar boron nucleus, the analysis of which confirms the ^1^ *J*_PPt_ coupling constant of around 3300 Hz.^18^

**Figure 2:**
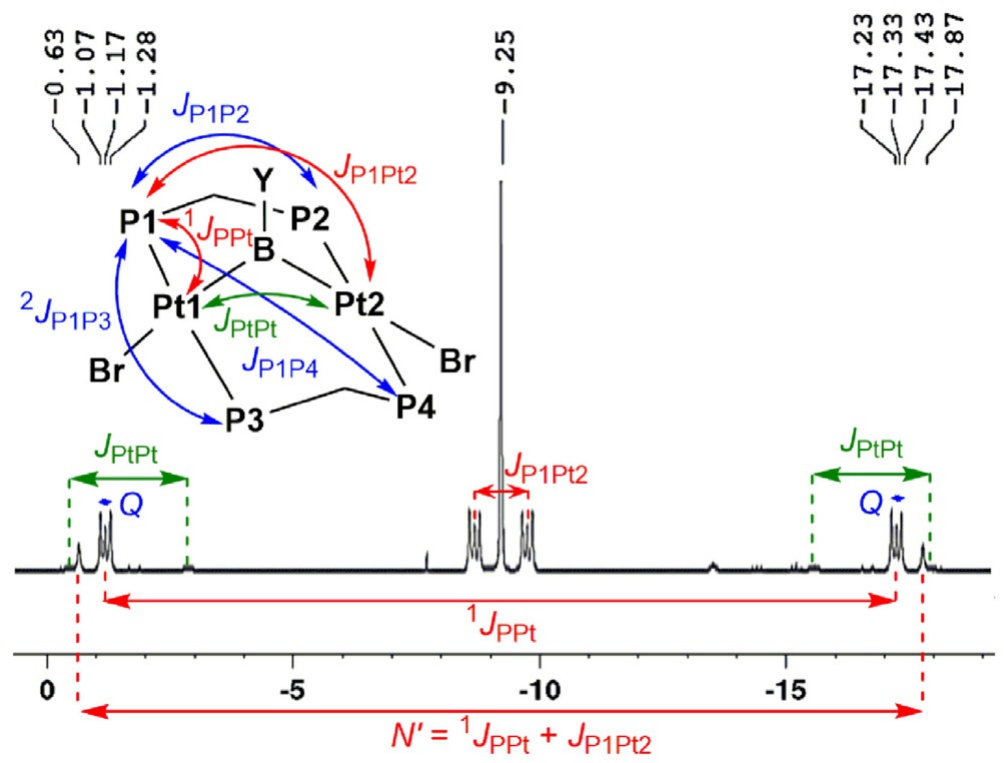
Annotated ^31^P{^1^H} NMR spectrum of **2‐Br**.

Figure 3 shows the solid‐state structures of the 32‐electron complexes **2‐Dur**, **2‐Br** and **2‐NMe_2_**, in which the Pt−Pt distances (3.1347(9) to 3.4015(4) Å) clearly indicate the absence of Pt−Pt bonding. This contrasts with the only other known boryl‐bridged (*μ*‐dppm)_2_Pt_2_ A‐frame complexes, [*μ*‐BCat{(*μ*‐dppm)_2_Pt_2_(BCat)(PR_3_)}] (Cat=catecholate, PR_3_=PPh_3_, *κ*_1_‐dppm), isolated from the reaction of [(PPh_3_)_2_Pt(BCat)_2_] with excess dppm, in which the short Pt−Pt distance of 2.77 Å indicates Pt−Pt bonding.^19^ Complex **2‐Br** displays crystallographically imposed *C*_2_ symmetry, with the symmetry axis passing through B1 and bisecting the Pt1⋅⋅⋅Pt1′ vector, and virtually no distortion from an idealized A‐frame, with the Pt centers in near square‐planar environments and a P1‐Pt1‐Pt1′‐P2 torsion angle (−1.51(3)°) close to 0°. As the steric demands of the substituent at boron increase (Br<NMe_2_<Dur) the geometry of the A‐frame becomes increasingly distorted, with the P1‐Pt1‐Pt2‐P4 torsion angles reaching around 26° in **2‐Dur** (compare side‐views of **2‐Dur**, **2‐Br** and **2‐NMe_2_** in Figure 3). The Pt−Br bond length also increases in the order Y=Br<BNMe_2_<Dur, which is in line with the increase in *trans* effect previously determined for boryl ligands at square‐planar Pt^II^ complexes.^20^ The boron atoms in the complexes **2‐Y** are trigonal planar and the Pt−B bond lengths span the range from 1.967(3) (**2‐Br**) to 2.042(9) Å (**2‐NMe_2_**), which is within the range of known platinum‐containing bimetallic bridging borylene complexes (1.910(4) to 2.091(4) Å).^14^

**Figure 3:**
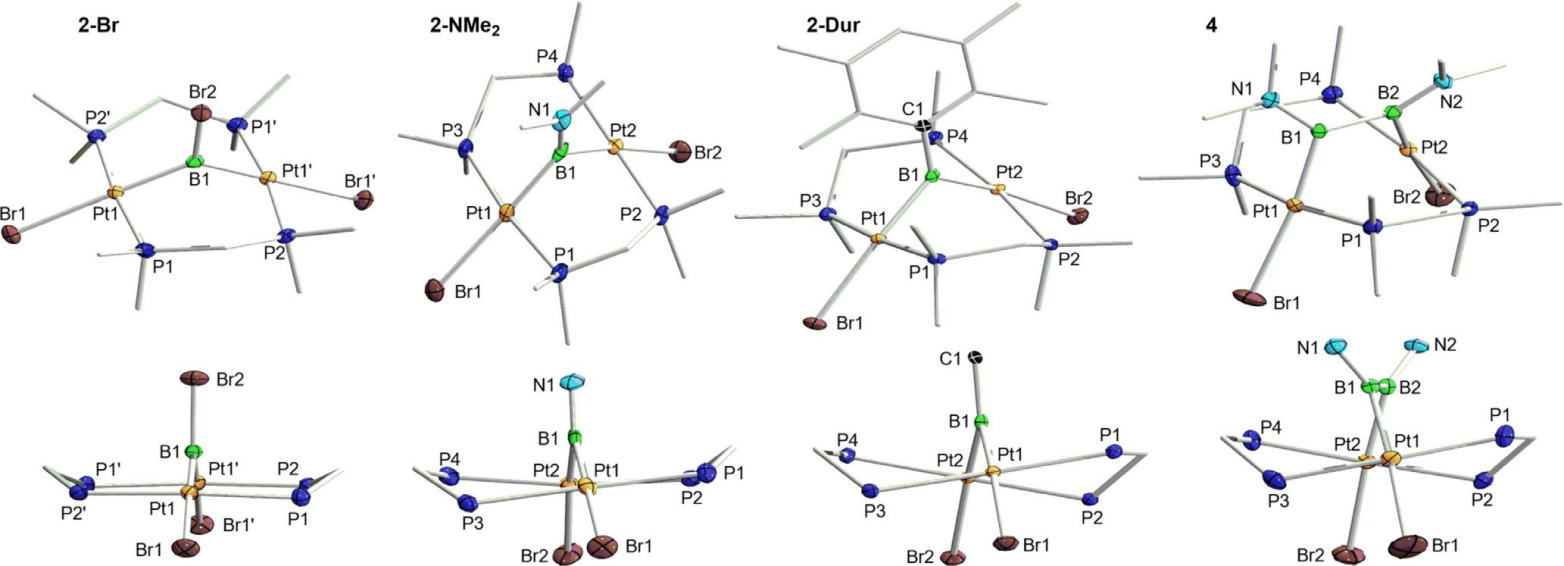
Top: Crystallographically derived molecular structures of (from left to right) **2‐Br**, **2‐NMe_2_**, **2‐Dur** and **4**. Thermal ellipsoids at 50 % probability. Thermal ellipsoids of ligand periphery and hydrogen atoms omitted for clarity. Bottom: Truncated side‐views of the same complexes evidencing the increasing distortion of the A‐frame through the increase of the P1/3‐Pt1‐Pt2‐P2/4 torsion angle. Thermal ellipsoids of ligand periphery and all hydrogen atoms omitted for clarity. Selected bond lengths (Å) and angles (°) for **2‐Br**: Pt1⋅⋅⋅Pt1′ 3.4015(4), Pt1−B1 1.967(3), Pt1−Br1 2.6069(4), Pt1‐B1‐Pt1′ 119.7(3), torsion angle P1‐Pt‐Pt1′‐Pt2 1.51(3) **| 2‐NMe_2_**: Pt1⋅⋅⋅Pt2 3.3003(4), Pt1−B1 2.028(10), Pt2−B1 2.042(9), B1−N1 1.395(12), Pt1−Br1 2.6298(9), Pt2−Br2 2.6236(9), Pt‐B1‐Pt2 108.4(5), torsion angles P1‐Pt1‐Pt2‐P2 4.96(7), P3‐Pt1‐Pt2‐P4 15.62(8) **| 2‐Dur**: Pt1⋅⋅⋅Pt2 3.1347(9), Pt1−B1 2.020(4), Pt2−B1 2.029(4), Pt1−Br1 2.6431(9), Pt2−Br2 2.6282(6), Pt‐B1‐Pt2 101.46(18), torsion angles P1‐Pt‐Pt2‐P2 26.59(3), P3‐Pt1‐Pt2‐P4 25.37(3) **| 4**: Pt1⋅⋅⋅Pt2 3.372(1), Pt1−B1 2.085(6), Pt2−B2 2.100(6), B1−B2 1.687(8), B1−N1 1.418(7), B2−N2 1.467(7), Pt1−Br1 2.6609(11), Pt2−Br2 2.6901(13), Pt1‐B1‐B2 112.5(4), Pt2‐B2‐B1 109.8(4), Σ∠_B1_ 359.1(4), Σ∠_B2_ 359.7(4), torsion angles P1‐Pt‐Pt2‐P2 27.29(6), P3‐Pt‐Pt2‐P4 27.60(6).

Given that the structural resemblance of **2‐Y** with *μ*‐borylene complexes contrasts with the unusually upfield‐shifted ^11^B NMR resonances, we analyzed the nature of the Pt−B bonding in **2‐Dur**, **2‐Br** and **2‐NMe_2_** with the computational EDA‐NOCV method.^21^ The calculations were carried out considering two distinct scenarios: 1) the interaction of the {(*μ*‐dmpm)Pt}_2_ and BY fragments in their electronic triplet spin states, corresponding to the formation of electron‐sharing Pt−B σ bonds in **2‐Y**, and 2) the interaction of both fragments in their electronic singlet states, corresponding to borylene‐type complexes with delocalized donor–acceptor bonding. For all **2‐Y** compounds studied herein the second scenario yields a significantly larger orbital interaction than the first (see Tables S1–S3 in the Supporting Information for details), the smaller orbital interaction values indicating a more appropriate choice of fragments.^22^ As a result, **2‐Y** should be regarded as bridging boranediyl (or dimetallaborane) rather than delocalized three‐center *μ*‐borylene complexes. In all cases, B−Pt bonding is dominated by electrostatic interactions (56 to 68 %), with non‐negligible contributions coming from orbital interactions. These result mainly from Δ*E*_orb(1)_ and Δ*E*_orb(2)_, that is, the electron‐sharing B−Pt σ‐bonds (Figure 4 b, c), while Δ*E*_orb(3)_ is related to π backdonation from the Pt atoms to boron (Figure 4 d), and accounts for merely 6 to 9 % of the orbital interaction stabilization. Therefore, in contrast to complexes **II** and **III** (Figure 1), there is no significant stabilization of the boron bridgehead through delocalized π backbonding in **2‐Y**.

**Figure 4:**
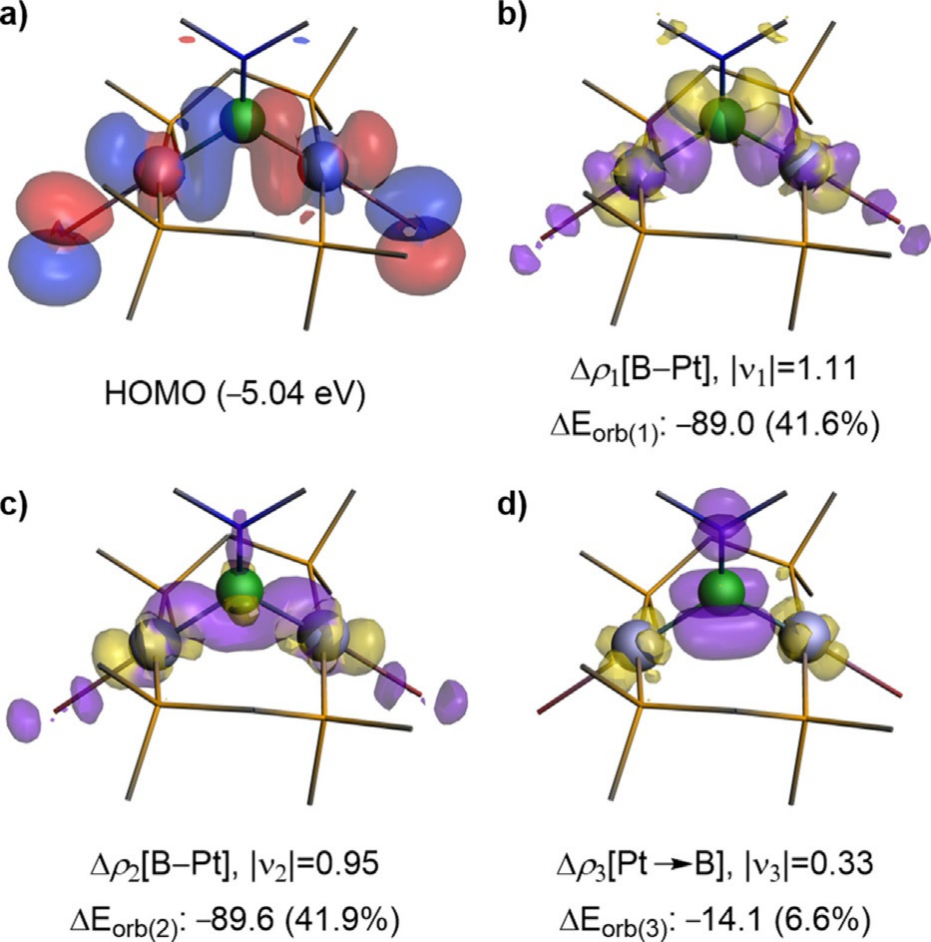
a) Plot of the HOMO of **2‐NMe_2_** at the B3LYP/TZV2P//ωB97XD/cc‐pVDZ,aug‐cc‐pVDZ‐PP{Pt} level of theory. b–d) Plot of deformation densities (Δρ_k_), at the same level of theory, of the main orbital interactions of **2‐NMe_2_** starting from fragments {(*μ*‐dmpm)Pt}_2_ and BY in their electronic triplet state. The |ν_k_| values correspond to the eigenvalues of the complementary eigenfunctions (ψ_−k_, ψ_k_) in the NOCV representation, while Δ*E*_orb(k)_ is the energy contribution (kcal mol^−1^) of the respective orbital interaction to the total orbital interaction energy, Δ*E*_orb_. The electron density flows from yellow to purple. Hydrogen atoms are omitted for clarity.

Whereas many *μ*‐borylene complexes, especially aminoborylenes, tend to be very stable,^4^, ^23^ the boranediyls **2‐Y** slowly decomposed in solution, the only identifiable decomposition product being the 30‐electron [(*μ*‐dppm)_2_Pt_2_Br_2_] complex **3** (Scheme 3). The stability of **2‐Y** decreased in the order of Y=Br>Dur≫NMe_2_, that is, with the increasing electron‐donating nature of Y. While this decomposition occurs with formal loss of „BY“, no ^11^B NMR resonance of any decomposition by‐product was detected.^24^ This is reminiscent of the decomposition of boryl complexes *trans*‐[(Cy_3_P)_2_Pd(Br)(BR_2_)] into *trans*‐[(Cy_3_P)_2_PdBr_2_] and *trans*‐[(Cy_3_P)_2_Pd(Br)H], in which the fate of the boryl ligand and the origin of the palladium‐bound hydride remains unclear.^25^ In contrast, platinum boryl complexes tend to be very stable as the Pt−B bond is remarkably resistant even to hydrolysis.^26^

When treated with 1,2‐dihalodiboranes(4), Pt^0^ complexes are known to form diborane(4)yl complexes by (sometimes reversible) oxidative addition of the boron–halogen bond^27^ or bis(boryl) complexes by oxidative addition of the B−B bond.27b, 28 When attempting to synthesize diboron‐bridged complexes in a similar manner from the reaction of **1** with 1,2‐dihaloboranes(4), no reaction was observed with 1,2‐dibromo‐1,2‐diduryldiborane(4), while the reaction with B_2_Br_4_ resulted solely in the formation of **2‐Br** (Scheme 4 a). This is reminiscent of the syntheses of the first bridging borylene complexes from B_2_Cl_2_Y_2_ (Y=NMe_2_, *t*Bu) and two equivalents of K[(C_5_H_4_R)MnR′(CO)_2_] (R=H, Me; R′=H, SiMe_2_Ph), in which the second BY moiety was lost as the corresponding diborane(6) derivative (YBH_2_)_2_.^4^, ^29^ The reaction of **1** with B_2_Br_2_(NMe_2_)_2_, however, yielded a mixture of **2‐NMe_2_** (δ_11B_=52 ppm, δ_31P_=−5.6 ppm) and a new species, complex **4**, displaying a ^31^P NMR singlet at −13.1 ppm (^1^ *J*_PPt_=3383 Hz) and a single, very broad ^11^B NMR resonance at 58 ppm (fwmh≈2000 Hz). This is similar to the ^11^B NMR shift of 55.6 ppm displayed by [*μ*‐{B_2_(NMe_2_)_2_}{(PEt_3_)_2_PtI}_2_], which is obtained from the 1:2 molar addition of B_2_I_2_(NMe_2_)_2_ to [Pt(PEt_3_)_2_],27a and suggests that **4** is also a *μ*‐bridging diborane(4)‐1,2‐diyl complex (Scheme 4 b).

**Scheme 4:**
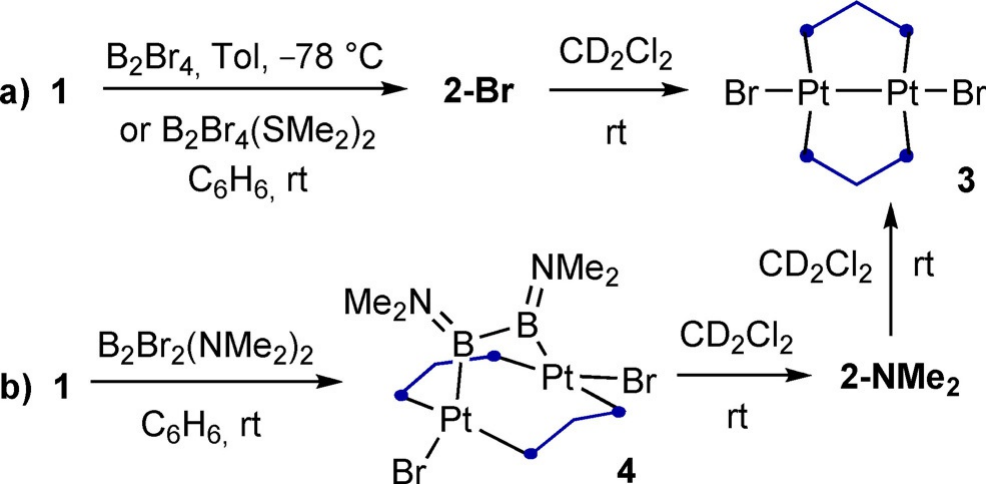
Reactions of **1** with diboranes(4). Tol=toluene.

This was confirmed by the X‐ray crystallographic analysis of colorless single crystals of **4** (Figure 3), the structure of which exhibits two platinum centers bridged by the *cis*‐1,2‐diaminodiborane(4)‐1,2‐diyl unit. The Pt1⋅⋅⋅Pt2 distance of **4** (3.372(1) Å) is only 0.07 Å longer than that of **2‐NMe_2_** (3.3003(4) Å) and is actually shorter than that of **2‐Br** (3.4015(4) Å) despite the additional aminoboryl unit in the bridge. The complex displays a strong distortion of its A‐frame geometry, with P1‐Pt1‐Pt2‐P2 and P3‐Pt1‐Pt2‐P4 torsion angles of around 27° (see side‐view of **4** in Figure 3). It is noteworthy that, whereas the B−B bond in [*μ*‐{B_2_(NMe_2_)_2_}{(PEt_3_)_2_PtI}_2_] (1.783(7) Å)27a is significantly longer than that of the B_2_I_2_(NMe_2_)_2_ precursor (1.684(6) Å),27d the B−B bond in **4** (1.687(8) Å) is of a similar length to that in B_2_Br_2_(NMe_2_)_2_ (1.682(16) Å),^30^ the elongation likely being prevented by the geometry imposed by the A‐frame. Like complexes **2‐Y** complex **4** proved very unstable in solution and in the solid state, decomposing to **2‐NMe_2_** and ultimately to **3**.^31^ Since [*μ*‐{B_2_(NMe_2_)_2_}{(PEt_3_)_2_PtI}_2_] did not display any sign of decomposition in solution up to 100 °C,27a we surmise that the instability of **4**, and presumably also of **2‐Y**, is caused by the strain of the A‐frame geometry rather than an intrinsic instability of diplatinum (di)boranediyls.

The *μ*‐(diborane‐1,2‐diyl) character of **4** is also supported by EDA‐NOCV calculations (see Table S4 and Figure S25 in the Supporting Information). Similar to **2‐Y**, the Δ*E*_orb_ term indicates a preference of electron‐sharing over donor‐acceptor Pt−B bonds. The bonding is again dominated by electrostatic interactions (66.5 %, see Supporting Information), with negligible π backdonation (Δ*E*_orb(4)_=3.4 % of the orbital interaction stabilization) from Pt to B.

It is noteworthy that the formation of **2‐Y** and **4** involves the highly selective, simultaneous oxidative *trans*‐addition of a B−Br bond at each of the two Pt^0^ centers. Computational analyses by Zeng and Sakaki on the mechanism of oxidative *trans*‐addition of BBr_2_(OSiMe_3_) to Pt(PMe_3_)_2_ have shown that, following pre‐coordination of the electron‐deficient borane to the electron‐rich Pt^0^ center, the addition of the B−Br bond may occur through one of two pathways: a) dissociation of Br^−^ to form a tightly bound [Pt(PMe_3_)_2_{BBr(OSiMe_3_)}]^+^[Br]^−^ ion pair, followed by direct nucleophilic attack of Br^−^ at the *trans* position, or b) oxidative *cis* addition, followed by thermal, rate‐limiting *cis*‐*trans* isomerization, the latter being slightly less favorable.^32^ While it is likely that a similar mechanism takes place here, further computational studies beyond the scope of this communication would be required to determine whether this occurs in a fully concerted or stepwise manner, following either pathway a) or b).

To conclude, we have isolated a series of diplatinum boranediyl and diborane‐1,2‐diyl A‐frame complexes, all of which proved highly unstable and prone to loss of the boron bridge. DFT calculations showed that the metal–boron bonds in these complexes are electron‐sharing σ bonds, with negligible contribution coming from π backdonation. This is in stark contrast with structurally related bridging borylenes, which display delocalized metal‐to‐boron π backbonding. Having thus established that the double oxidative addition of boron–halogen bonds at two tethered low‐valent metal centers provides a reliable synthetic platform for accessing these rare dimetallated (di)boranes, we expect that variations in the nature of the metal centers and/or bridging ligand framework should yield in future, more stable, (di)boranediyls, the reactivity of which remains to be discovered.

## Experimental Section

**Crystallographic data**: Deposition numbers 1986723, 1986724, 1986725, 1986726, 1986727, and 1986728 contain the supplementary crystallographic data for this paper. These data are provided free of charge by the joint Cambridge Crystallographic Data Centre and Fachinformationszentrum Karlsruhe Access Structures service.

## Conflict of interest

The authors declare no conflict of interest.

## Supporting information

As a service to our authors and readers, this journal provides supporting information supplied by the authors. Such materials are peer reviewed and may be re‐organized for online delivery, but are not copy‐edited or typeset. Technical support issues arising from supporting information (other than missing files) should be addressed to the authors.

Supplementary
