# Supplementary material for: Boranediyl‐ and Diborane(4)‐1,2‐diyl‐Bridged Platinum A‐Frame Complexes
Source: Chemistry. 2020 Jun 17;26(39):8518–23. doi: 10.1002/chem.202001168 (PMC7384048; doi:10.1002/chem.202001168)
Supplement: Supplementary file 1 — Supplementary [file CHEM-26-8518-s001.pdf]

Chemistry–A European Journal

Supporting Information

Boranediyl- and Diborane(4)-1,2-diyl-Bridged Platinum A-Frame Complexes

Carina Brunecker,[a] Jonas H. Müssig,[a] Merle Arrowsmith,[a] Felipe Fantuzzi,[a, b] Andreas Stoy,[a] Julian Böhnke,[a] Alexander Hofmann,[a] Rüdiger Bertermann,[a] Bernd Engels,[b] and Holger Braunschweig*[a]

Table of contents

Methods and materials S2
Synthetic procedures S3
NMR spectra of new compounds S6
Decomposition of **4** S17
X-ray crystallographic data S18
Computational details S21
References S37

Methods and materials

All manipulations were performed either under an atmosphere of dry argon or *in vacuo* using standard Schlenk line or glovebox techniques. Deuterated solvents were dried over molecular sieves and degassed by three freeze-pump-thaw cycles prior to use. All other solvents were distilled and degassed from appropriate drying agents. Solvents (both deuterated and non-deuterated) were stored under argon over activated 4 Å molecular sieves. NMR spectra were acquired on a Bruker Avance 500 NMR spectrometer (^{1}H and $^{1}H\{^{31}P\}$: 500.1 MHz, $^{11}B\{^{1}H\}$: 160.5 MHz, $^{13}C\{^{1}H\}$: 125.8 MHz, $^{31}P\{^{1}H\}$: 202.5 MHz), Bruker Avance 400 NMR spectrometer (^{1}H: 400.1 MHz, $^{11}B\{^{1}H\}$: 128.4 MHz, $^{13}C\{^{1}H\}$: 100.6 MHz, $^{31}P\{^{1}H\}$: 162.0 MHz), a Av NEO 400 SB spectrometer ($^{195}Pt\{^{1}H\}$: 86.0 MHz). Chemical shifts (δ) are given in ppm and internally referenced to the carbon nuclei ($^{13}C\{^{1}H\}$) or residual protons (^{1}H) of the solvent. $^{11}B\{^{1}H\}$, $^{31}P\{^{1}H\}$ and $^{195}Pt\{^{1}H\}$ NMR spectra were referenced to [$BF_3 \cdot OEt_2$], 85% H_3PO_4 or Na_2[$PtCl_6$] as an external standard. Microanalyses (C, H, N) were performed on an Elementar vario MICRO cube elemental analyzer.

Solvents and reagents were were purchased from Sigma Aldrich or Alfa Aesar. [Pt(nbe)$_3$] (nbe = norbornene),[1] BBr_2Dur (Dur = 2,3,5,6-Me_4C_6H),[2] and $B_2Br_2(NMe_2)_2$,[3] were synthesized using literature procedures or based on literature procedures. BBr_2NMe_2 was obtained as the by-product of the synthesis of $B_2Br_2(NMe_2)_2$ and isolated by fractional distillation.

Synthetic procedures

[{(μ-dmpm)Pt(nbe)}$_2$], 1

[Pt(nbe)$_3$] (2.00 g, 4.19 mmol) was dissolved in benzene (10 mL) and dmpm (= bis(dimethylphosphino)methane, 570 mg, 4.19 mmol) was added at 0 °C. The reaction mixture was stirred for 1 h at 0 °C and for 1 h at rt. The resulting precipitate was filtered off and washed with benzene (3 x 1 mL) to yield **2** (1.53 g, 1.80 mmol, 86% based on Pt) as a beige solid. ^{1}H NMR (400.1 MHz, C_6D_6): δ = 2.90 + 2.85 (two br. m, 2H each, PC*H$_2$*), 2.36 + 2.33 (two s + satellites, 2H each, $^2J_{H\text{-}Pt}$ = 60.7, 58.0 Hz, nbe-*H*C=C*H*), 1.77-1.98 (m, 6H, nbe-C*H* + nbe-C*H$_2$*), 1.49-1.59 (m, 12H, nbe-C*H$_2$* + PC*H$_3$*), 1.30-1.41 (m, 14H, bridging C*H$_2$* + PC*H$_3$*), 1.19 (m + satellites, 6H, $^3J_{H\text{-}Pt}$ = 27.8 Hz, PC*H*$_3$), 0.5 (tm, 2H, $^3J_{H\text{-}H}$ = 9.2 Hz, bridging C*H$_2$*) ppm. Elemental analysis (%) calculated for [$C_{24}H_{48}P_4Pt_2$] (M_W = 850.7): C 33.89, H 5.69; found: C 34.11, H 5.77.

[{μ-(BDur)}{(μ-dmpm)PtBr}$_2$], 2-Dur

In a vial, one molar equivalent of BBr_2Dur (5.36 mg, 17.6 µmol) was added to a benzene solution (700 µL) of **1** (15.0 mg, 17.6 µmol). The mixture was shaken, causing the immediate precipitation of an orange solid, which after filtration was recrystallized from CH_2Cl_2/pentane to yield analytically pure **2-Dur** (11.2 mg, 11.6 µmol, 66%). ^{1}H NMR (400.1 MHz, CD_2Cl_2): δ = 7.25 (s, 1H, Dur-C*H*.), 3.20 (s, 6H, Dur-C*H*$_3$), 2.28 (s, 6H, Dur-C*H*$_3$), 1.81-1.86 (m, 2H, PC*H*$_2$), 1.93 (br. + satellites, 12H, $^3J_{H\text{-}Pt}$ = 30.6 Hz, PC*H*$_3$), 1.44-1.35 (br. + satellites, 12H, $^3J_{H\text{-}Pt}$ = 38.0 Hz, PC*H*$_3$), 1.30-1.80 (m, 2H, PC*H*$_2$) ppm. ^{13}C{^{1}H}NMR (100.6 MHz, CD_2Cl_2): δ = 148.9 (br, B*C*$_{Dur}$), 136.9 (*m*-Dur-*C*), 135.2 (*o*-Dur-*C*H), 135.0 (*p*-Dur), 30.2-29.8 (m, P*C*H$_2$), 25.1 (Dur-*C*H$_3$), 21.3 (Dur-*C*H$_3$), 15.9-15.6 (m + satellites, P*C*H$_3$), 14.9-14.7 (m + satellites, P*C*H$_3$) ppm. *Note: the ^{13}C NMR resonances of the dmpm ligands appear as complex multiplets due to coupling with the various ^{31}P nuclei of the molecules.* ^{31}P{^{1}H} NMR (202.5 MHz, CD_2Cl_2): δ = −13.6 (s + higher order satellites, $^1J_{Pt1\text{-}P1}$ = 3517 Hz, $J_{Pt2\text{-}P1}$ = 241 Hz, $Q = J_{P1\text{-}P2} + J_{P1\text{-}P3}$ = 56 Hz) ppm. ^{11}B{^{1}H} NMR (160.5 MHz, CD_2Cl_2): δ = 98 (br s, , fwmh ≈ 2150 Hz) ppm. Elemental analysis (%) calculated for [$C_{20}H_{41}BBr_2P_4Pt_2$] (M_W = 966.2): C 24.86, H 4.28; found: C 25.28, H 4.48.

[{μ-(BBr)}{(μ-dmpm)PtBr}$_2$], 2-Br

Path a) In a vial, one molar equivalent of a 0.09 M solution of Br_3B in toluene (4.42 mg, 17.7 µmol) was added to a benzene solution (700 µL) of **1** (15.0 mg, 17.7 µmol). The mixture

was shaken, causing the immediate precipitation of a yellow solid, which after filtration was recrystallized from CH_2Cl_2/pentane to yield analytically pure **2-Br** (5.40 mg, 5.92 μmol, 34%). **Path b)** In a vial, one molar equivalent of $Me_2S{\cdot}BBr_3$ (7.70 mg, 23.5 μmol) was added to a benzene solution (0.7 mL) of **1** (7.70 mg, 23.5 μmol). The mixture was shaken, causing the immediate precipitation of a yellow solid, which after filtration was recrystallized from CH_2Cl_2/pentane to yield analytically pure **2-Br** (20.8 mg, 22.8 μmol, 97%). ^{1}H NMR (500.1 MHz, CD_2Cl_2): δ = 1.91-1.96 (m, 2H, PCH_2), 1.88 (dm, 2H, $^2J_{H\text{-}H}$ = 14.1 Hz, PCH_2), 1.77 (m + satellites, 12H, $^3J_{H\text{-}Pt}$ = 30.0 Hz, PCH_3), 1.67 (br. m + satellites, 12H, $^3J_{H\text{-}Pt}$ = 34.6 Hz, PCH_3) ppm. ^{13}C{^{1}H} NMR (125.8 MHz, CD_2Cl_2): δ = 26.0 (t + satellites, $^1J_{C\text{-}P}$ = 16.4 Hz, $^3J_{C\text{-}Pt}$ = 43.9 Hz, P*C*H_2), 14.7-15.3 (m + satellites, P*C*H_3) ppm. ^{31}P{^{1}H} NMR (202.5 MHz, CD_2Cl_2): δ = –9.3 (s + higher order satellites, $^1J_{Pt1\text{-}P1}$ = 3272 Hz, $J_{Pt2\text{-}P1}$ = 219 Hz, $J_{Pt\text{-}Pt}$ = 510 Hz, *Q* = $J_{P1\text{-}P2}$ + $J_{P1\text{-}P3}$ = 42 Hz) ppm. ^{11}B{^{1}H} NMR (128.5 MHz, CD_2Cl_2): δ = 85 (br s, fwmh ≈ 2000 Hz) ppm. ^{195}Pt{^{1}H} NMR (86.1 MHz, CD_2Cl_2): δ = −3863 (br. m, $^1J_{Pt\text{-}P}$ ≈ 3300 Hz) ppm. Elemental analysis (%) calculated for [$C_{10}H_{28}BBr_3P_4Pt_2$] (M_W = 909.8): C 13.16, H 3.09; found: C 13.39, H 3.21.

[{*μ*-(BNMe$_2$)}{(*μ*-dmpm)PtBr}$_2$], 2-NMe$_2$

In a vial one equivalent of $BBr_2(NMe_2)$ (5.05 mg, 23.5 μmol) was added to a benzene solution (0.7 mL) of **1** (20.0 mg, 23.5 μmol). The mixture was shaken, causing the immediate precipitation of an orange solid, which after filtration was recrystallized from CH_2Cl_2/pentane to yield a handful of single crystals of **2-NMe$_2$** suitable for X-ray crystallographic analysis. ^{11}B{^{1}H} NMR (160.5 MHz, CD_2Cl_2): δ = 52 (br s, fwmh ≈ 1200 Hz) ppm. ^{31}P{^{1}H} NMR (202.5 MHz, CD_2Cl_2): δ = −5.6 (s + higher order satellites, $^1J_{Pt1\text{-}P1}$ = 3353 Hz, $J_{Pt2\text{-}P1}$ = 220 Hz, *Q* = $J_{P1\text{-}P2}$ + $J_{P1\text{-}P3}$ = 39 Hz) ppm. *Note: Due to rapid decomposition in solution,* ***2-NMe$_2$*** *could not be fully characterized. The ^{11}B and ^{31}P NMR spectra provided in Fig. S12 and Fig. S13 were obtained from the crude product immediately after completion of the reaction. Single crystals of* ***[(μ-dmpm)PtBr]$_2$*** *(3, δ(^{31}P) = –23.4 ppm) were repeatedly isolated as the sole identifiable decomposition product (see Scheme 3 in the manuscript for the solid-state structure of* ***3****).*

[(μ-dmpm)PtBr]$_2$, 3

Small crop of crystals isolated from the decomposition of **2-NMe$_2$**. ^{1}H NMR (400.6 MHz, CD_2Cl_2): δ = 2.85 (tm + satellites, $^2J_{H\text{-}P}$ = 20.6 Hz, $^3J_{H\text{-}Pt}$ = 20.9 Hz, 4H, PCH_2), 1.78 (br. m + satellites, 24H, $^3J_{H\text{-}Pt}$ = 29.4 Hz, PCH_3) ppm. ^{13}C{^{1}H} NMR (100.7 MHz, CD_2Cl_2): δ = 41.0 (t + satellites, $^1J_{C\text{-}P}$ = 17 Hz, $^3J_{C\text{-}Pt}$ = 52 Hz, P*C*H_2), 16.8-17.5 (m + satellites, P*C*H_3) ppm. ^{31}P{^{1}H}

NMR (162.2 MHz, CD_2Cl_2): δ = –23.4 (s + higher order satellites, $^{1}J_{Pt1\text{-}P1}$ = 2640 Hz, $J_{Pt2\text{-}P1}$ = 151 Hz, $Q = J_{P1\text{-}P2} + J_{P1\text{-}P3}$ = 74.5 Hz) ppm.

[{*μ*-($BNMe_2$)$_2$}{(*μ*-dmpm)PtBr}$_2$], 4

In a vial, one molar equivalent of $B_2Br_2(NMe_2)_2$ (6.34 mg, 23.5 µmol) was added to a benzene solution (0.7 mL) of **1** (20.0 mg, 23.5 µmol). The mixture was shaken, causing the immediate precipitation of an orange solid, which after filtration was recrystallized from CH_2Cl_2/pentane to yield analytically pure **4** (15.3 mg, 16.1 µmol, 91%). ^{1}H NMR (400.1 MHz, CD_2Cl_2): δ = 3.20 (s, 6H, NC*H*$_3$), 2.85 (s, 6H, NC*H*$_3$), 2.03-2.09 (m, 4H, PC*H*$_2$), 1.95 (br + satellites, 12H, $^{3}J_{H\text{-}Pt}$ = 26.7 Hz, PC*H*$_3$), 1.52 (br + satellites, 12H, $^{3}J_{H\text{-}Pt}$ = 45.4 Hz, PC*H*$_3$), ppm. ^{13}C{^{1}H} NMR (100.6 MHz, CD_2Cl_2): δ = 47.6 (s + satellites, $^{3}J_{Pt\text{-}C}$ = 98 Hz, N*C*H$_3$), 45.9 (s + satellites, $^{3}J_{Pt\text{-}C}$ = 64 Hz, NC*H*$_3$), 32.7 (t + satellites, $^{1}J_{C\text{-}P}$ = 15.3 Hz, $^{2}J_{C\text{-}Pt}$ = 39.2 Hz, P*C*H$_2$), 16.9-17.5 (m, P*C*H$_3$), 16.5 (br + satellites, $^{2}J_{C\text{-}Pt}$ = 38.2 Hz, P*C*H$_3$) ppm. ^{31}P{^{1}H} NMR (162.0 MHz, CD_2Cl_2): δ = –13.1 (br s + satellites, $^{1}J_{Pt\text{-}P}$ = 3383 Hz) ppm. ^{11}B{^{1}H} NMR (128.5 MHz, CD_2Cl_2): δ = 58 (br s, fwmh ≈ 2000 Hz) ppm. *Note: Due to rapid decomposition in solution and in the solid state, NMR spectra had to be recorded immediately after completion of the reaction. Figure S21 showcases the decomposition of an analytically pure sample of* ***4*** *into* ***2-NMe₂*** *and ultimately* ***3*** *in solution at rt over 3 days. Multiple attempts at elemental analysis yielded results fitting mixtures of* ***4*** *and* ***2-NMe$_2$*** *in varying ratios*. Example: Elemental analysis (%) calculated for [$C_{14}H_{40}B_2Br_2N_2P_4Pt_2$] ($M_W$ = 931.98): C 18.04, H 4.33, N 3.01; found: C 17.52, H 4.19, N 2.05. *Note: these values fit a ca. 1:1 mixture of* ***4*** *and* ***2-NMe$_2$*** *(calcd. C 17.26, H 4.12, N 2.32).*

NMR spectra of new compounds

Figure S1. ^{1}H NMR spectrum of **1** in C_6D_6.

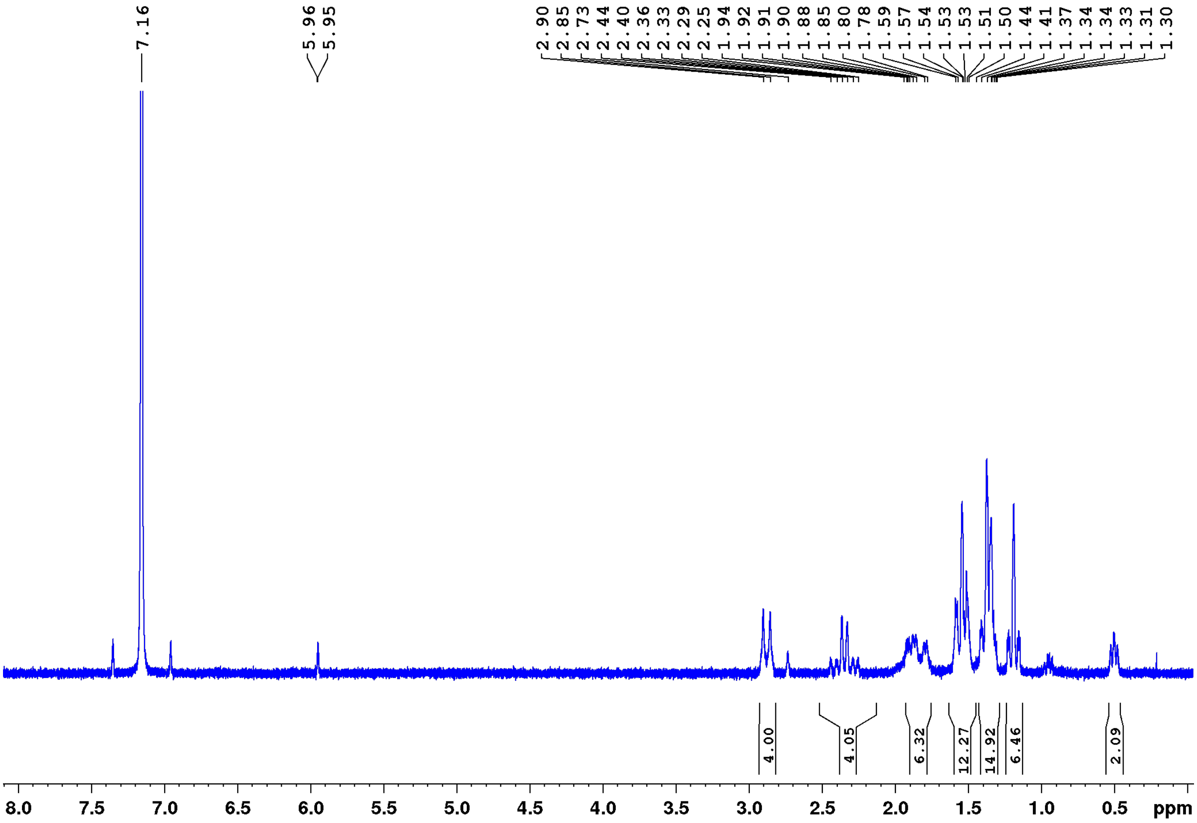

Figure S2. $^{31}P\{^{1}H\}$ NMR spectrum of **1** in C_6D_6.

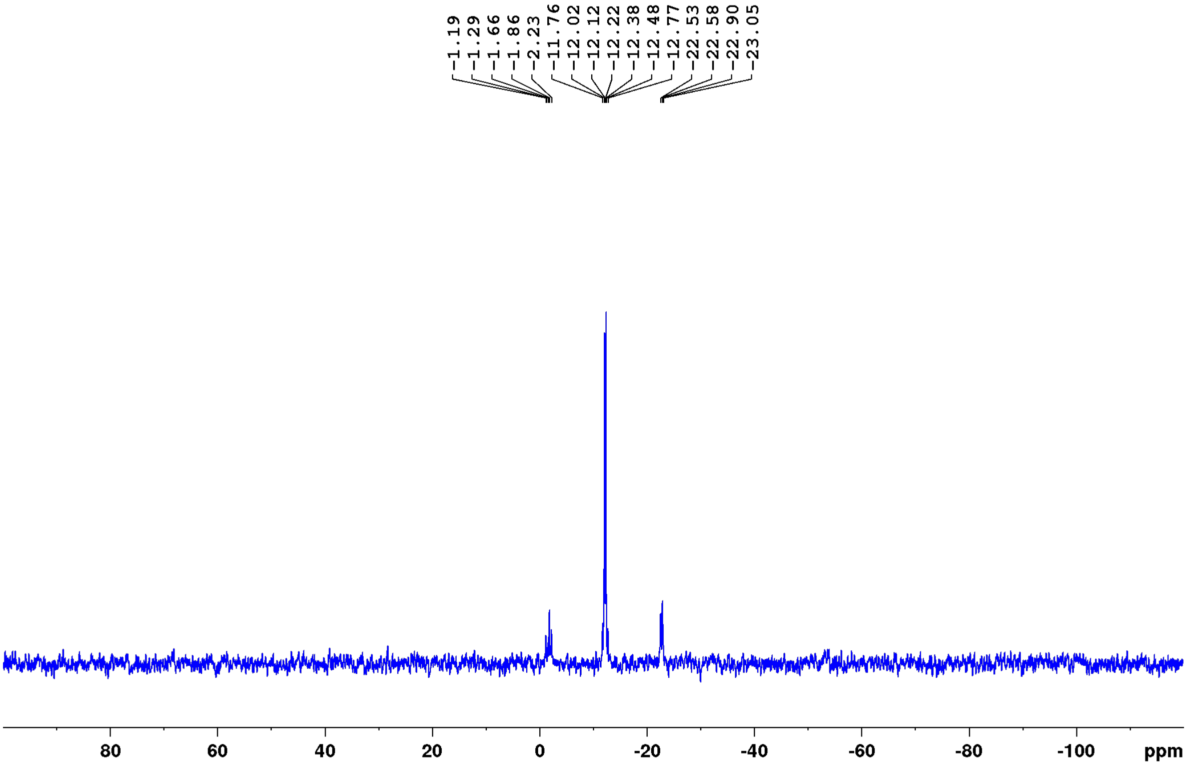

Note: *The following isolated A-frame borane- and diboranediyl complexes were not indefinitely stable in solution and decomposed rapidly under reduced pressure, which is why NMR spectra of isolated samples may contain significant amounts of residual crystallization solvent.*

Figure S3. ^{1}H NMR spectrum of **2-Dur** in CD_2Cl_2. The additional resonance at 0.88 ppm corresponds to residual pentane.

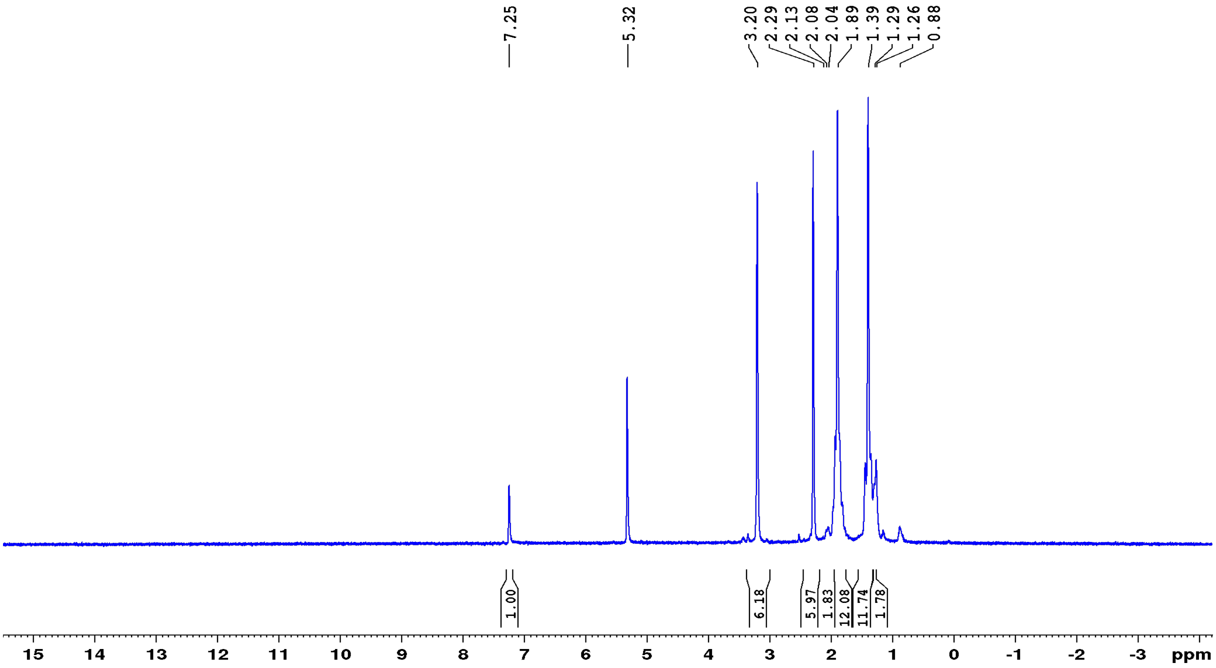

Figure S4. ^{13}C{^{1}H} NMR spectrum of **2-Dur** in CD_2Cl_2. The additional resonance at 14.2 ppm corresponds to residual pentane.

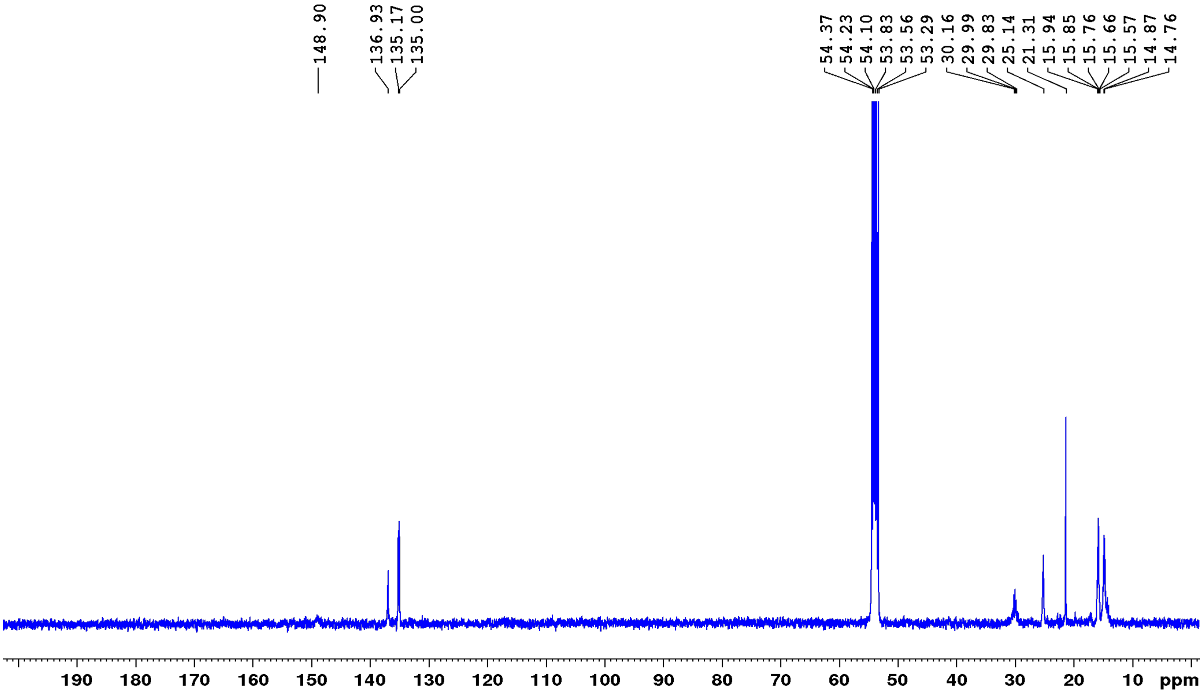

Figure S5. $^{11}B\{^{1}H\}$ NMR spectrum of **2-Dur** in CD_2Cl_2.

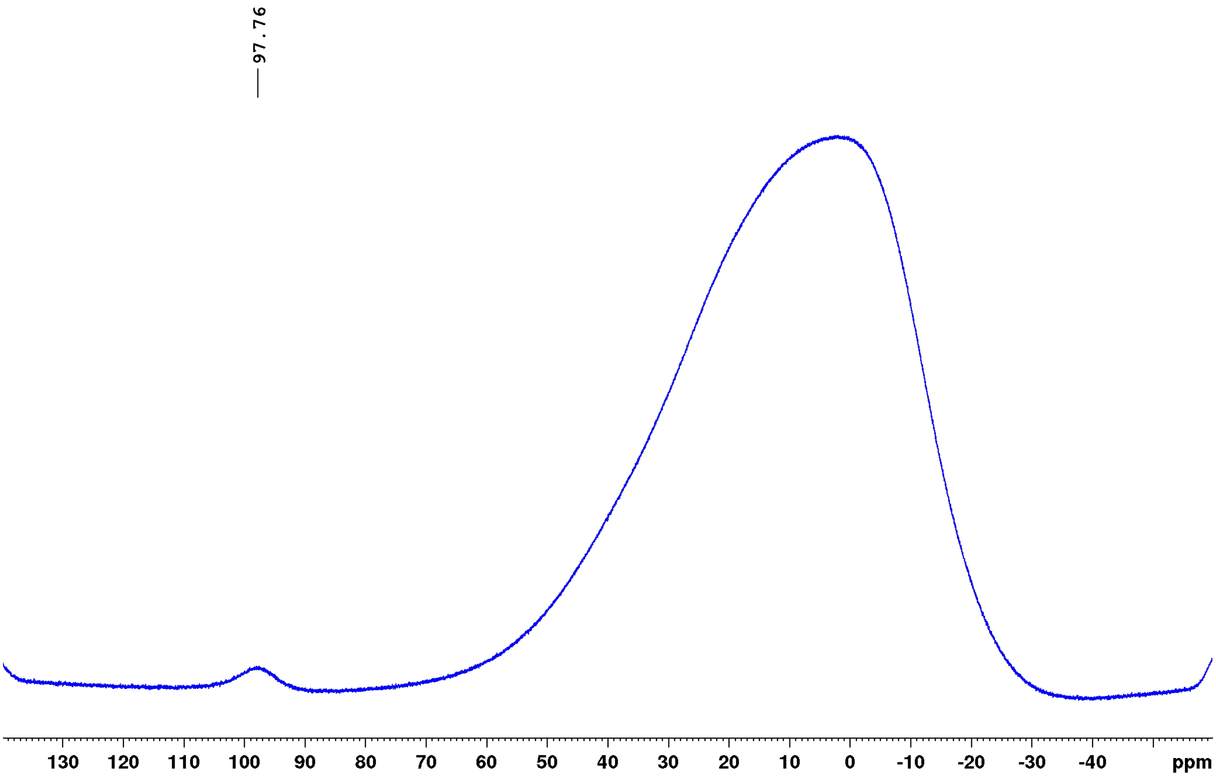

Figure S6. $^{31}P\{^{1}H\}$ NMR spectrum of **2-Dur** in CD_2Cl_2.

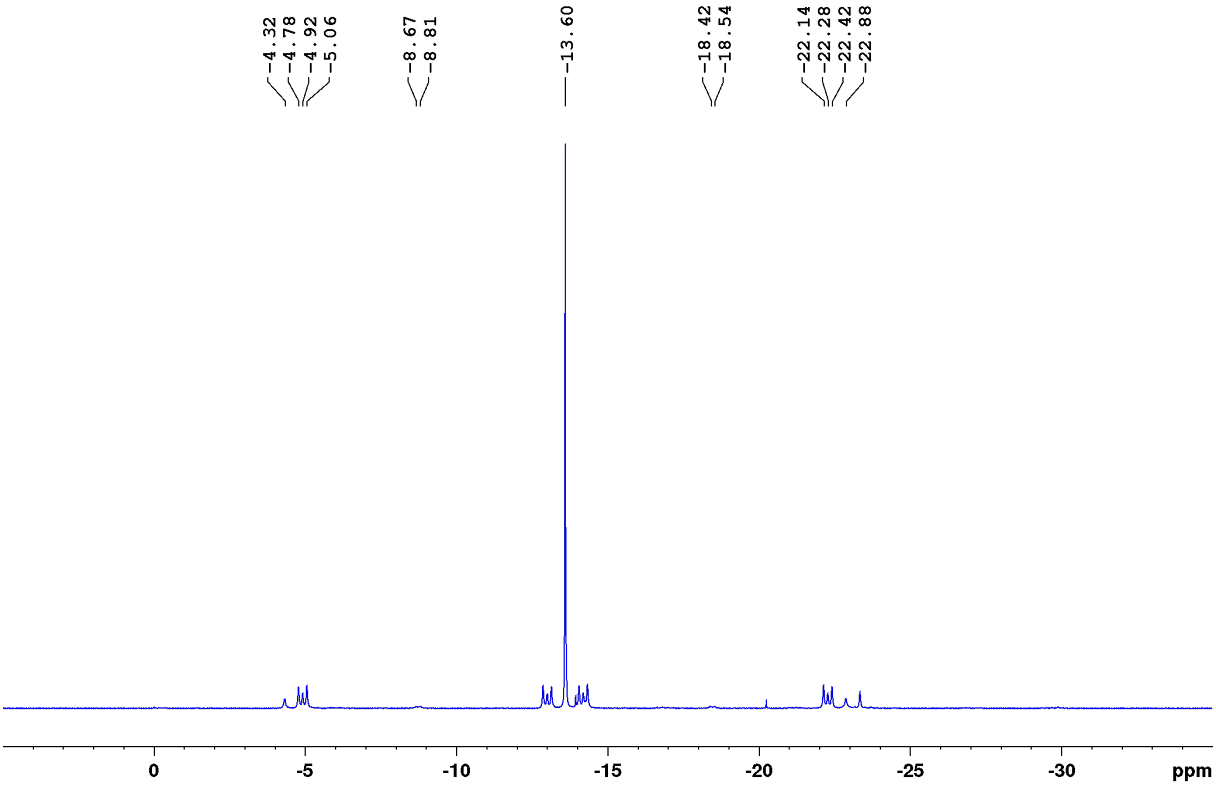

Figure S7. ^{1}H NMR spectrum of **2-Br** in CD_2Cl_2. The additional resonance at 7.35 ppm corresponds to residual benzene.

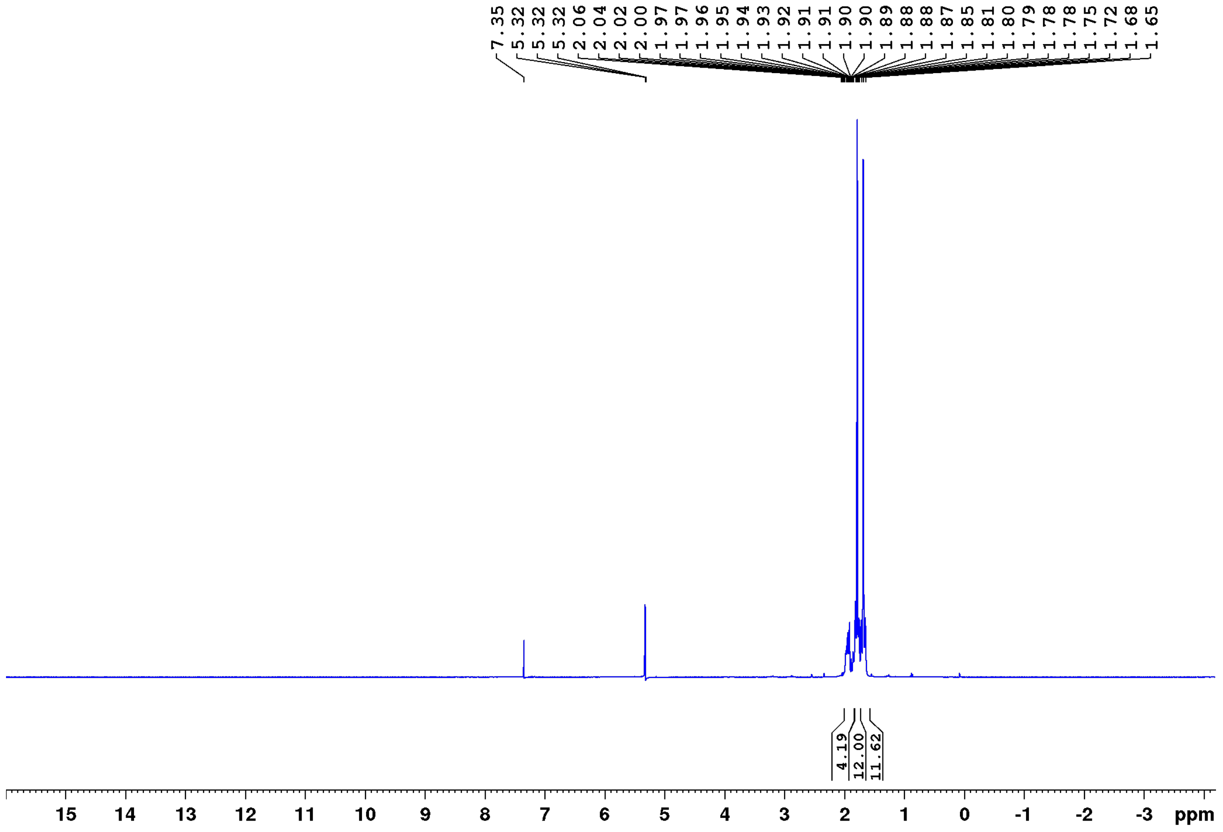

Figure S8. ^{13}C{^{1}H} NMR spectrum of **2-Br** in CD_2Cl_2. The additional resonance at 128.7 ppm corresponds to residual benzene.

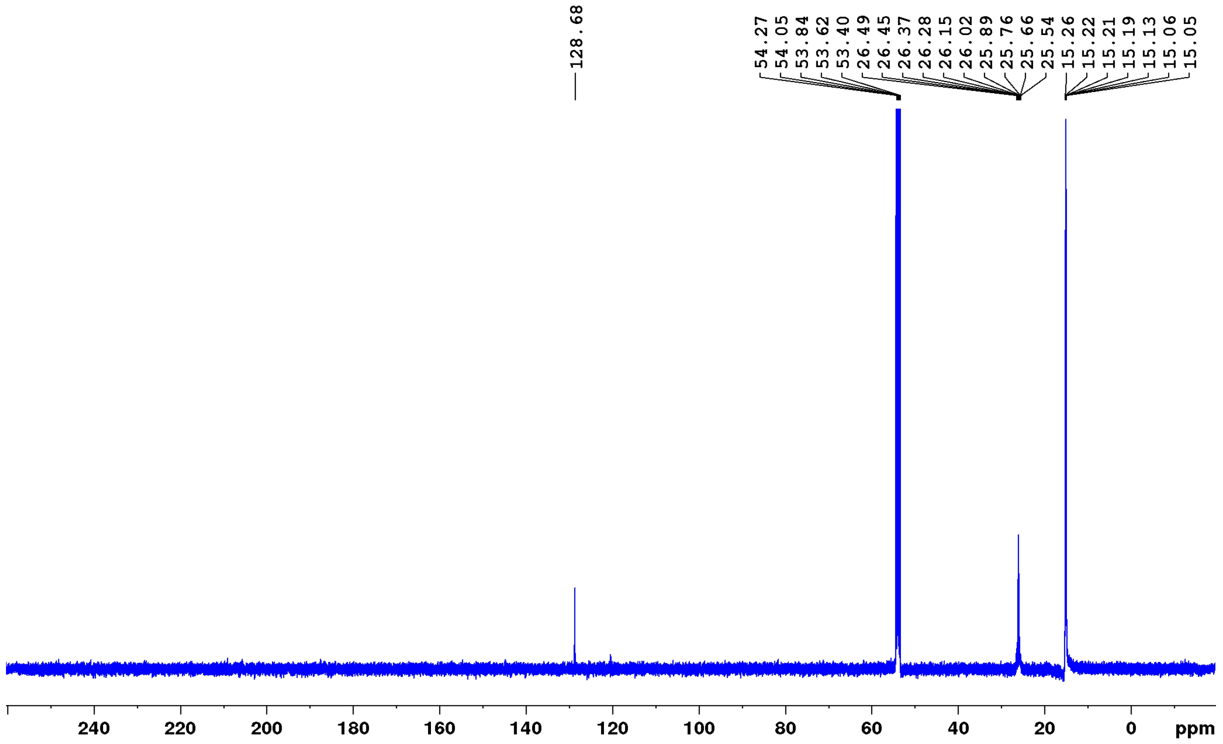

Figure S9. $^{11}B\{^1H\}$ NMR spectrum of **2-Br** in CD_2Cl_2.

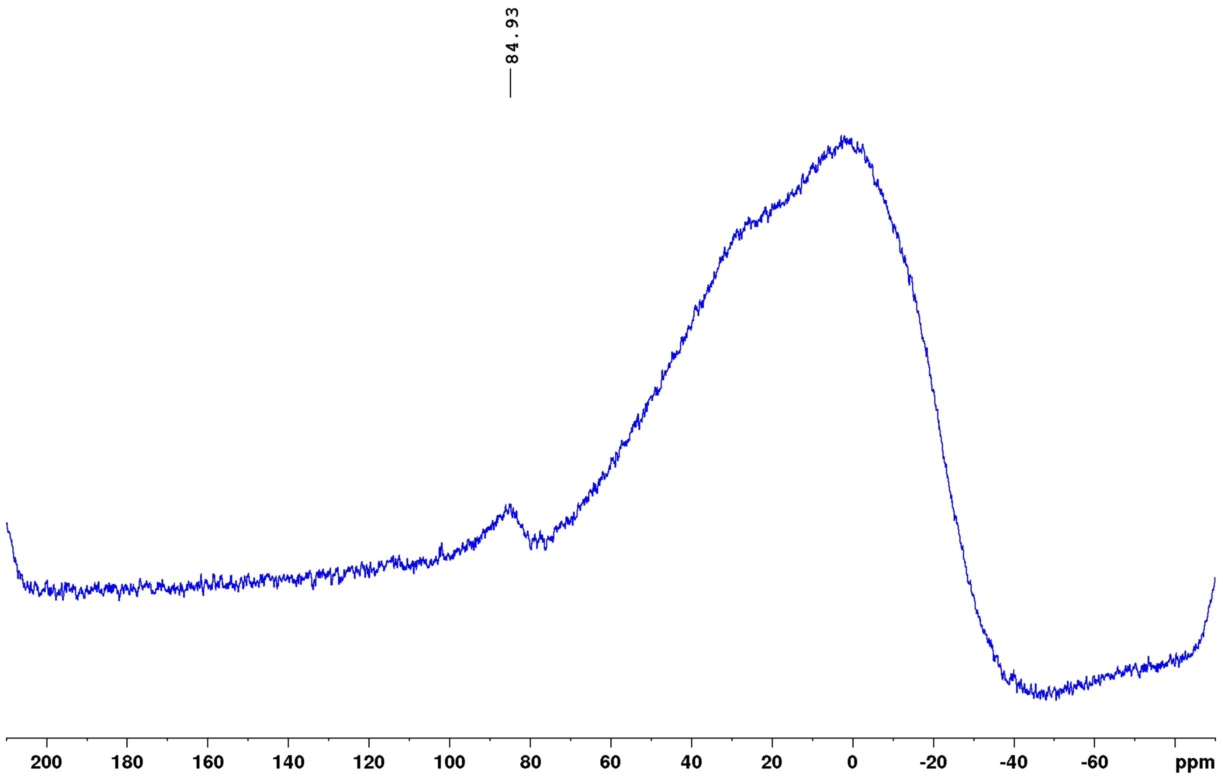

Figure S10. $^{31}P\{^1H\}$ NMR spectrum of **2-Br** in CD_2Cl_2.

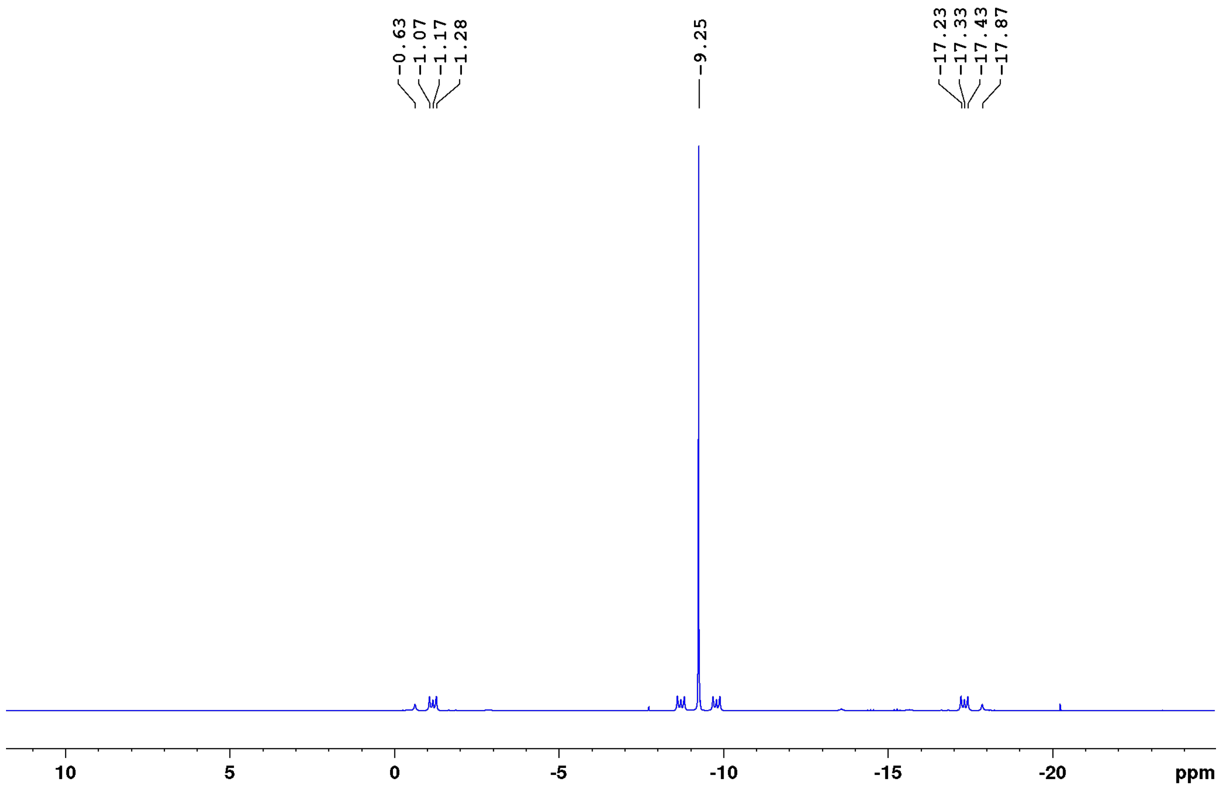

Figure S11. $^{195}Pt\{^{1}H\}$ NMR spectrum of **2-Br** in CD_2Cl_2.

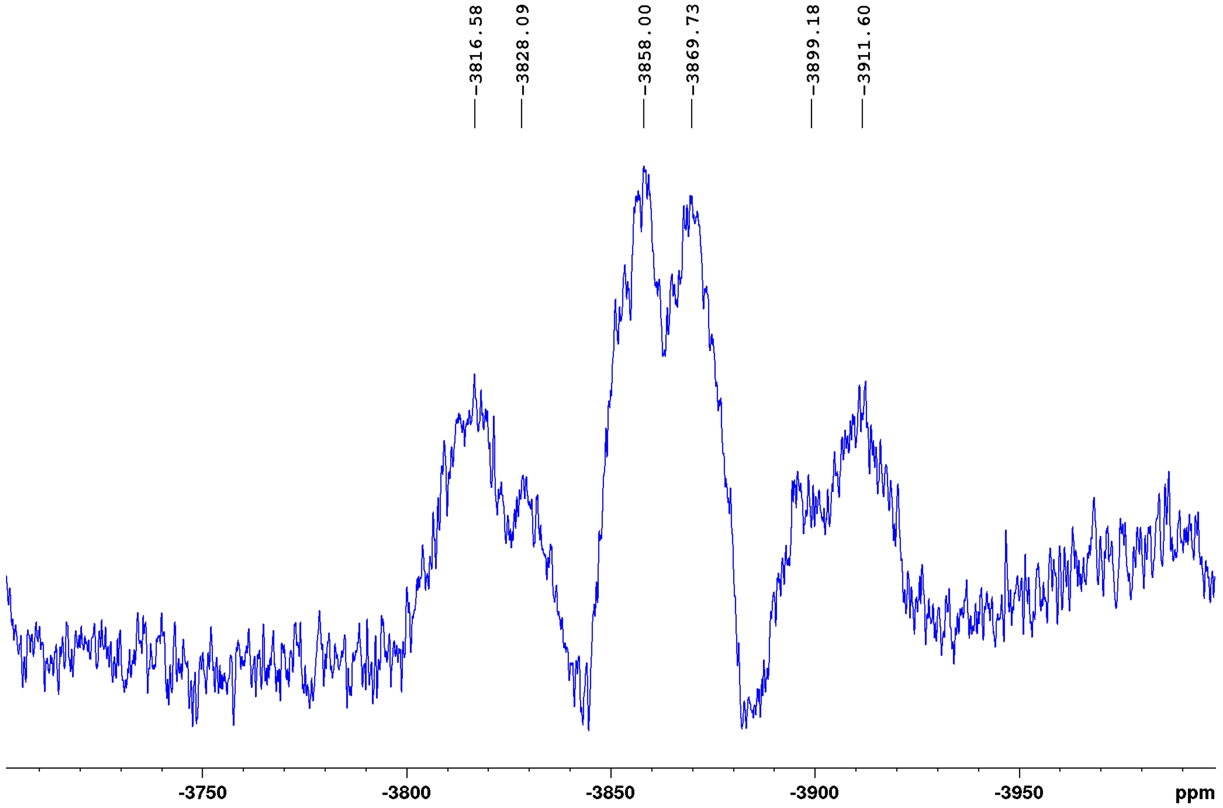

Figure S12. $^{11}B\{^1H\}$ NMR spectrum of the crude product from the synthesis of **2-NMe$_2$** in CD_2Cl_2. The additional small resonance at 28 ppm corresponds to unreacted $BBr_2(NMe_2)$.

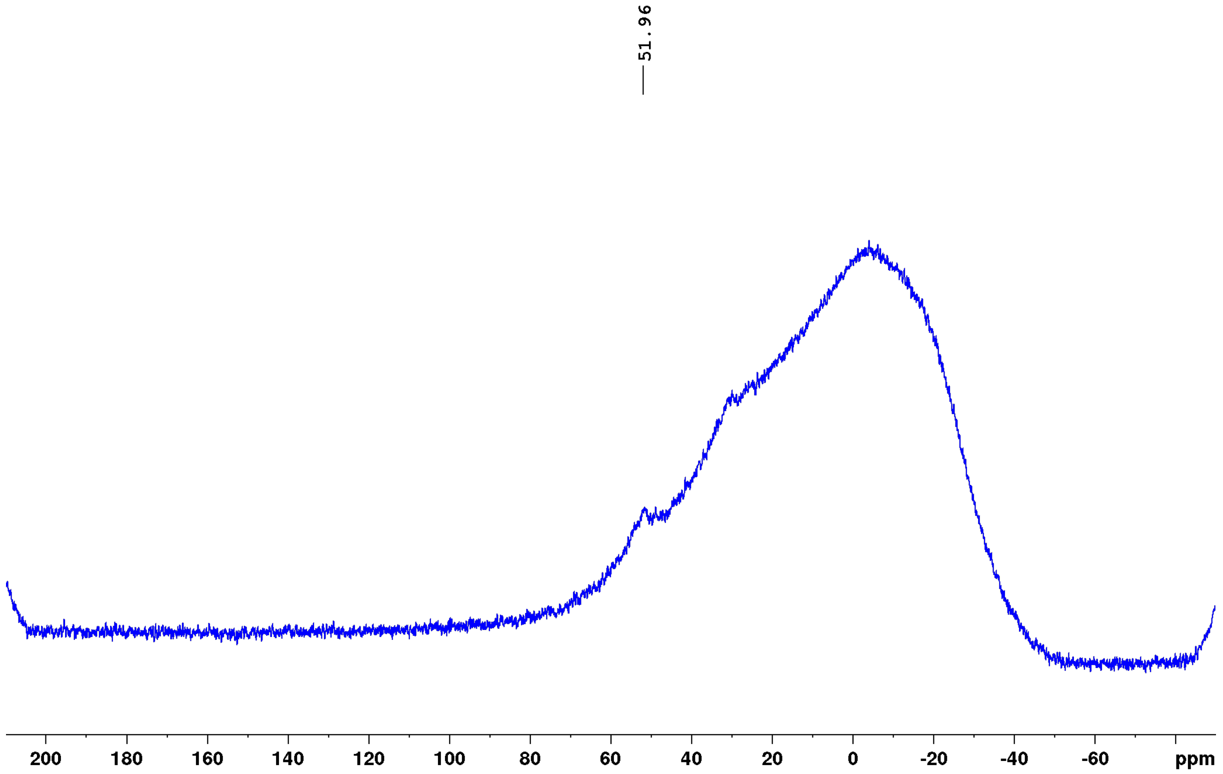

Figure S13. $^{31}P\{^1H\}$ NMR spectrum of the crude product from the synthesis of **2-NMe$_2$** in CD_2Cl_2. The additional resonance centered at −11 ppm corresponds to unreacted complex **1**.

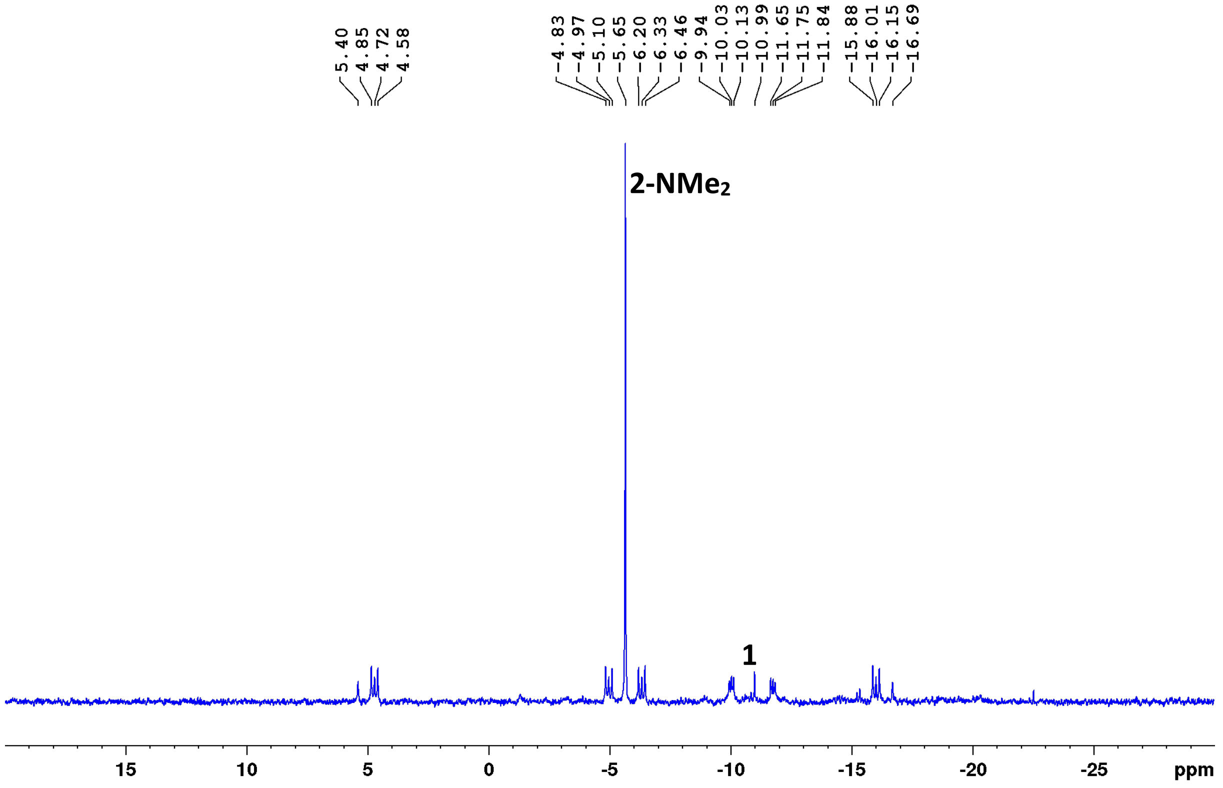

Figure S14. ^{1}H NMR spectrum of **3** in CD_2Cl_2. The additional resonances at 0.89, 1.30, and 7.35 ppm correspond to residual pentane and benzene.

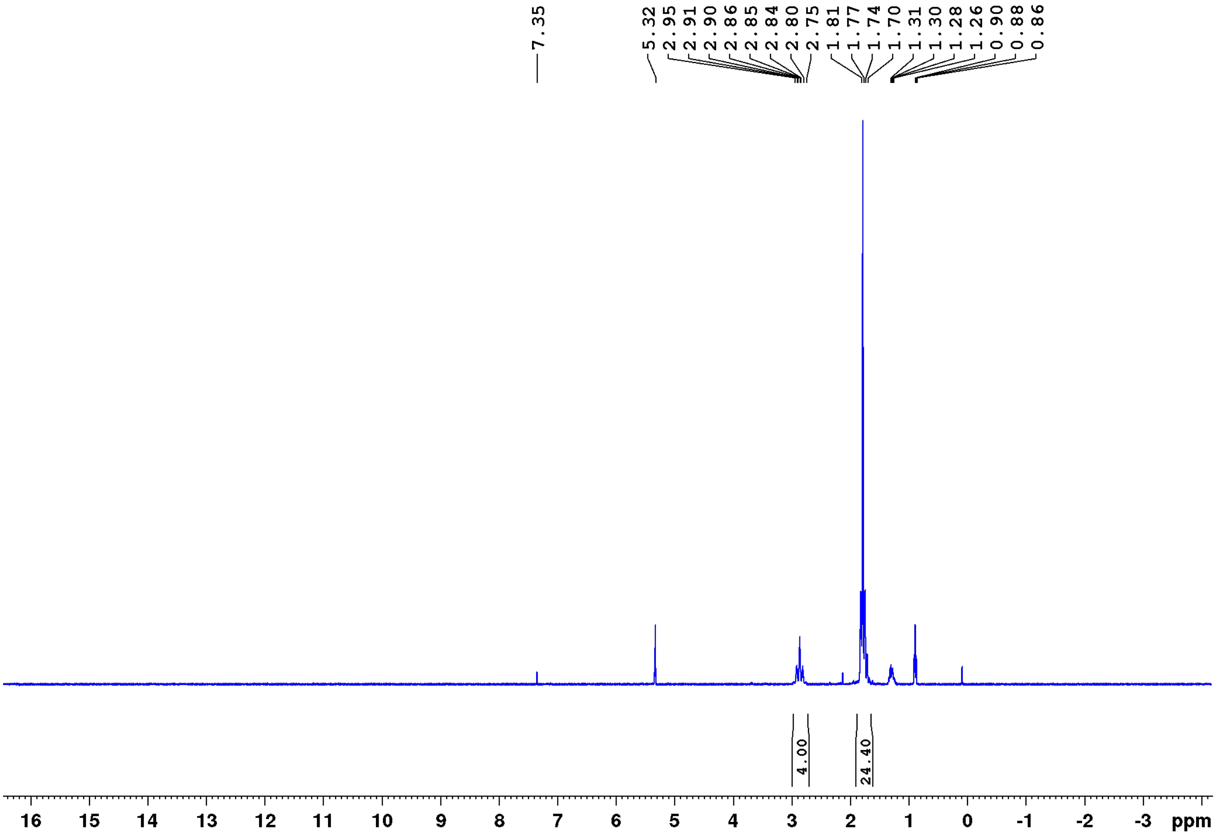

Figure S15. $^{13}C\{^{1}H\}$ NMR spectrum of **3** in CD_2Cl_2. The additional resonances at 14.2, 22.8 and 34.6 ppm correspond to residual pentane.

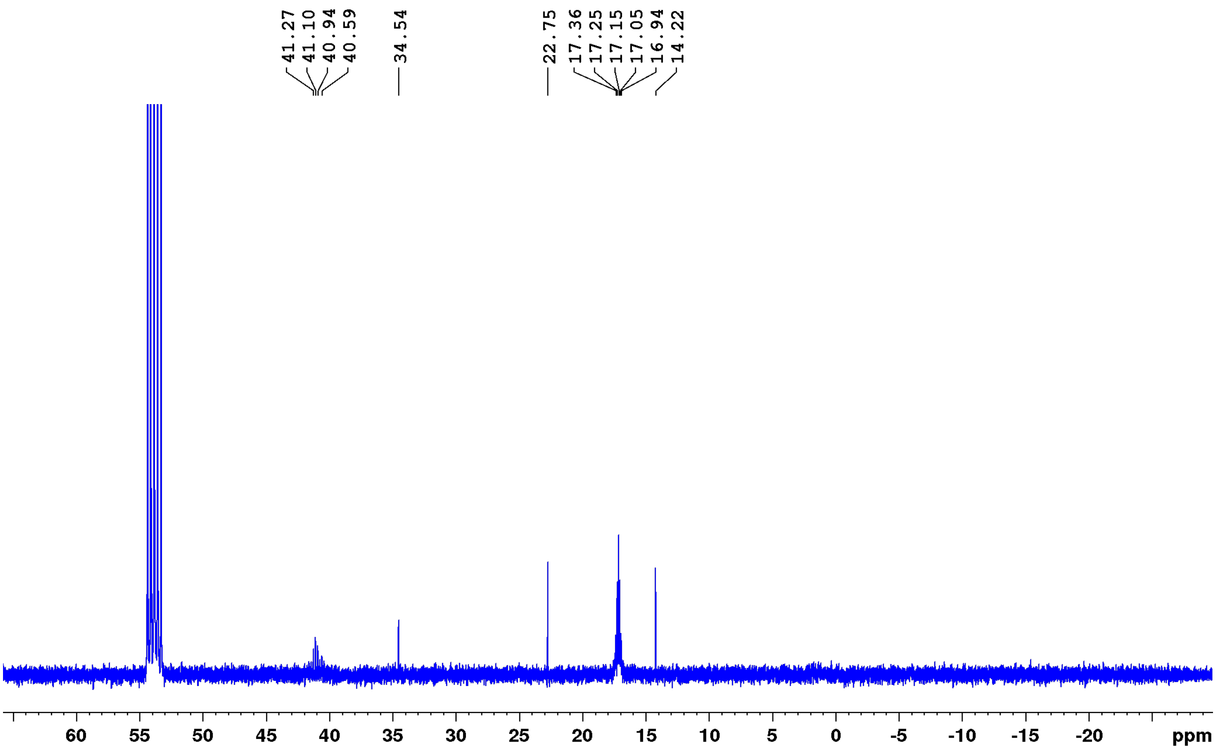

Figure S16. $^{31}P\{^{1}H\}$ NMR spectrum of **3** in CD_2Cl_2.

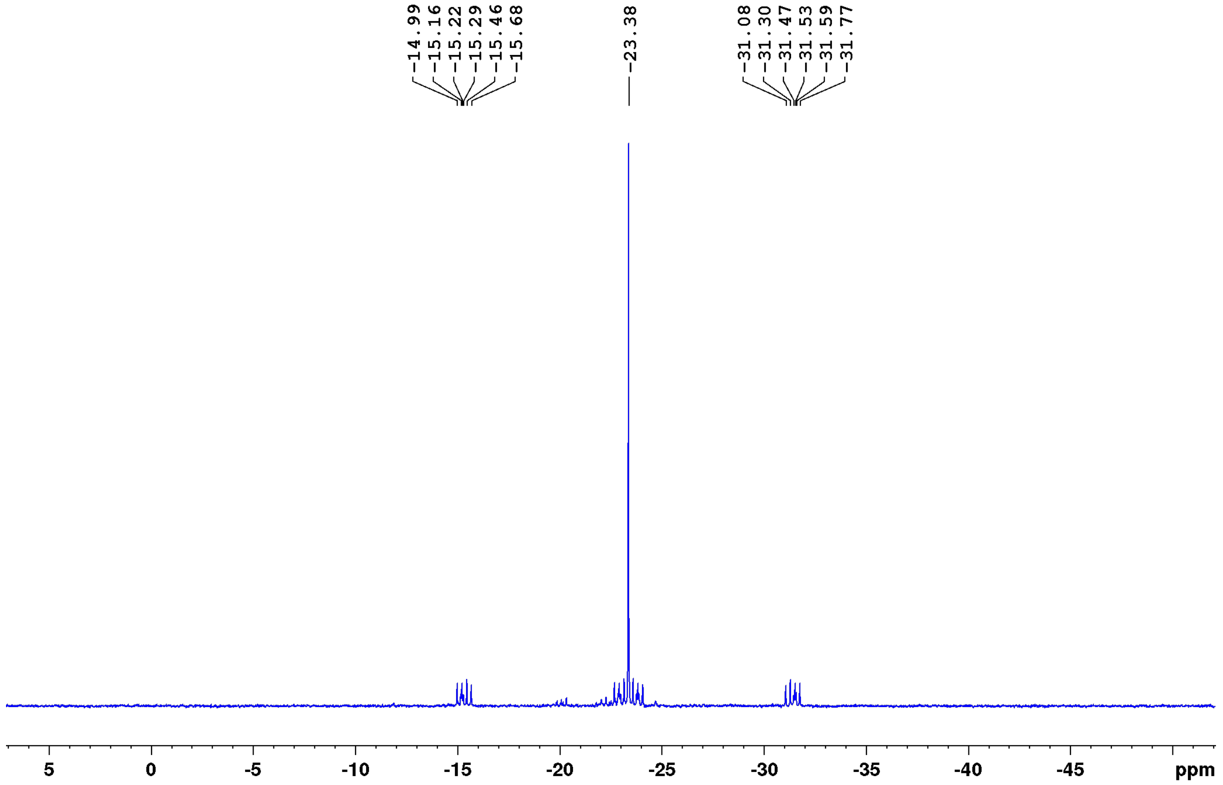

Figure S17. ^{1}H NMR spectrum of **4** in CD_2Cl_2.

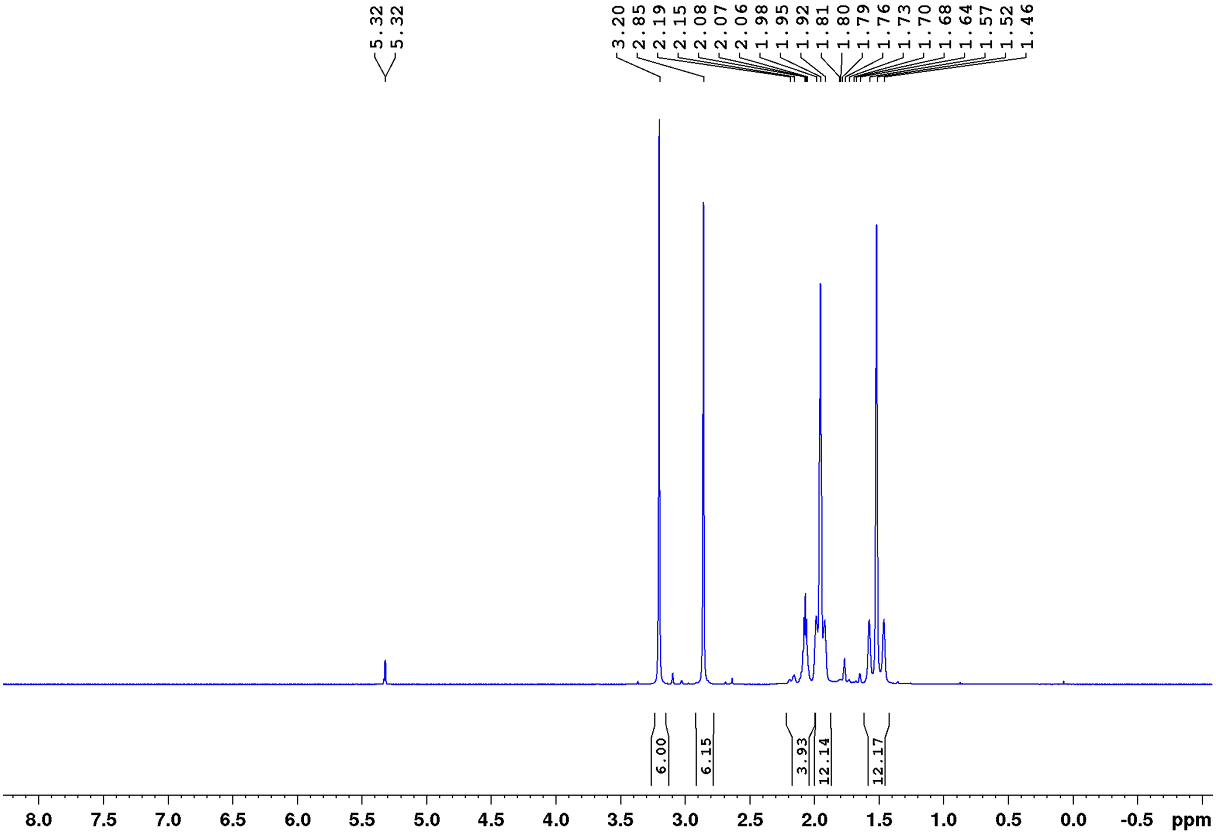

Figure S18. $^{13}C\{^{1}H\}$ NMR spectrum of **4** in CD_2Cl_2.

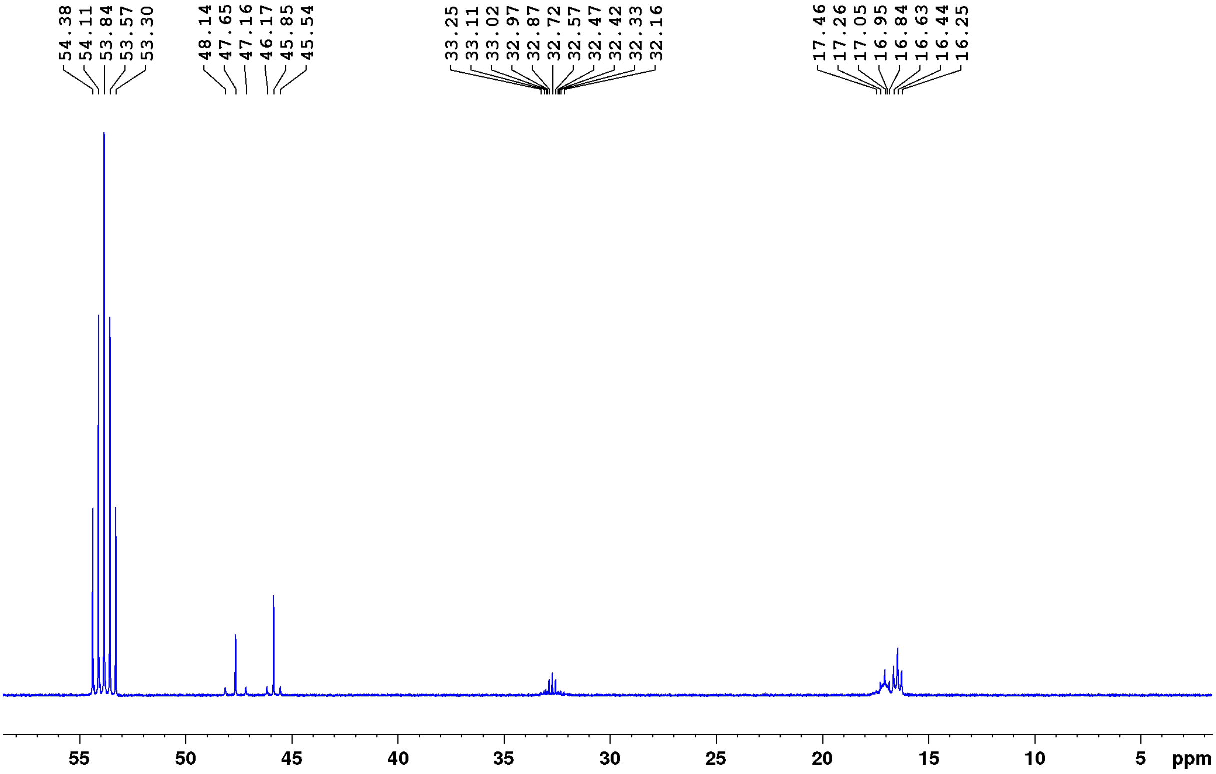

Figure S19. $^{11}B\{^1H\}$ NMR spectrum of **4** in CD_2Cl_2.

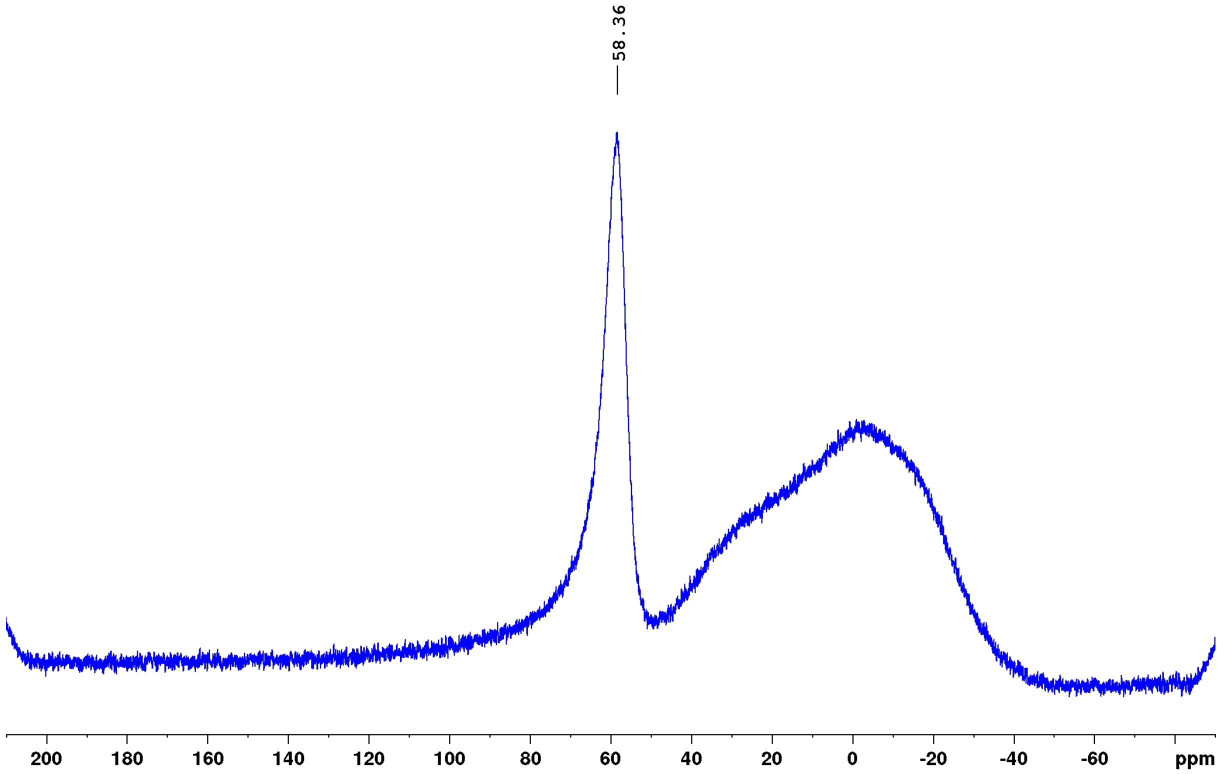

Figure S20. $^{31}P\{^1H\}$ NMR spectrum of **4** in CD_2Cl_2.

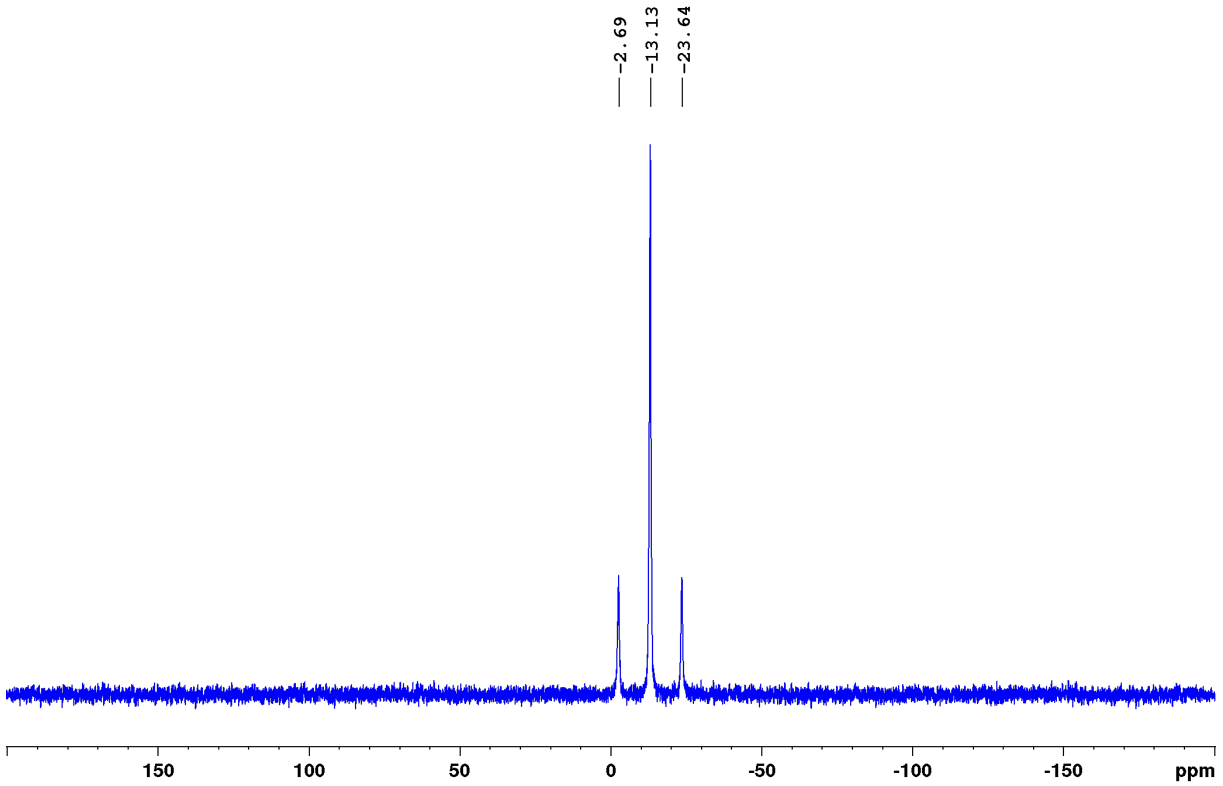

Decomposition of 4

Figure S21. Stack plot of $^{31}P\{^{1}H\}$ NMR spectra showing the decomposition of **4** (♦) into **2-NMe$_2$** (■) and ultimately **3** (●) in CD_2Cl_2 at rt over several days.

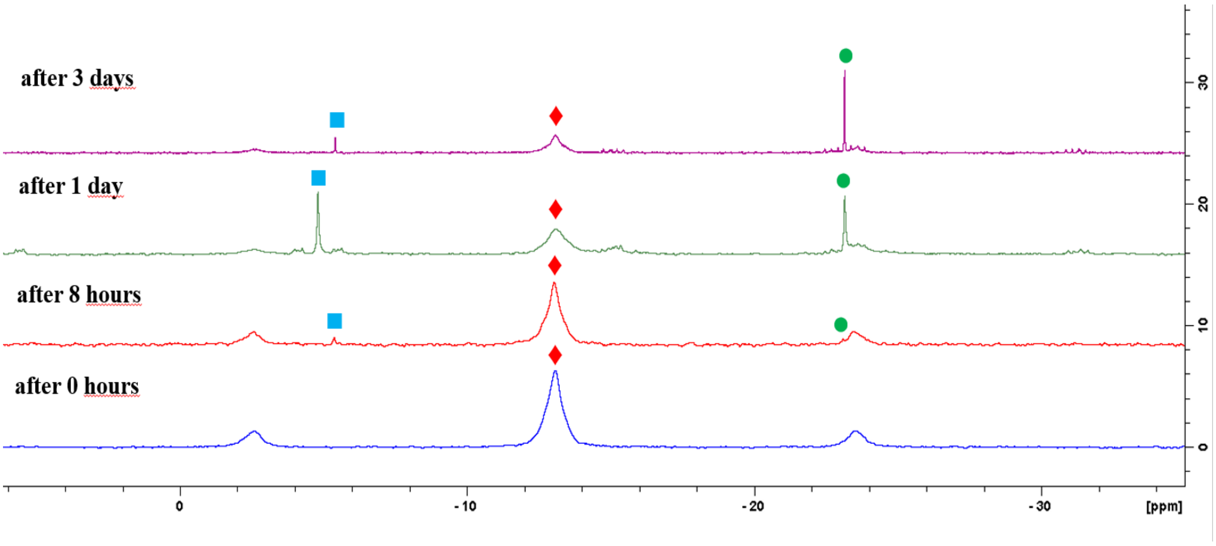

X-ray crystallographic data

Crystal data were collected on a BRUKER D8 QUEST diffractometer equipped with a CMOS area detector and multi-layer mirror monochromated $Mo_{K\alpha}$ radiation. Structures were solved using the intrinsic phasing method,[4] refined with the SHELXL program[5] and expanded using Fourier techniques. All non-hydrogen atoms were refined anisotropically. Hydrogen atoms were included in structure factor calculations. All hydrogen atoms were assigned to idealized geometric positions.

Crystallographic data have been deposited with the Cambridge Crystallographic Data Center as supplementary publication nos. CCDC 1986723–1986728. These data can be obtained free of charge from The Cambridge Crystallographic Data Centre *via* www.ccdc.cam.ac.uk/data_request/cif.

Crystal data for 1: $C_{24}H_{48}P_4Pt_2$, $M_r = 850.68$, colorless plate, 0.125×0.054×0.028 mm^3, triclinic space group $P\bar{1}$, $a = 8.8739(9)$ Å, $b = 9.400(4)$ Å, $c = 9.686(3)$ Å, $\alpha = 67.30(2)°$, $\beta = 89.698(12)°$, $\gamma = 71.177(10)°$, $V = 698.9(3)$ Å^3, $Z = 1$, $\rho_{calcd} = 2.021$ g·cm^{-3}, $\mu = 10.237$ mm^{-1}, $F(000) = 408$, $T = 100(2)$ K, $R_1 = 0.0151$, $wR^2 = 0.0349$, 2747 independent reflections [2θ≤52.032°] and 140 parameters.

Crystal data for 2-Dur: $C_{20}H_{41}BBr_2P_4Pt_2{\cdot}(C_6H_6)_{0.5}$, $M_r = 1005.27$, yellow block, 0.284×0.22×0.078 mm^3, orthorhombic space group *Pbca*, $a = 17.209(5)$ Å, $b = 11.805(3)$ Å, $c = 31.175(12)$ Å, $V = 6333(3)$ Å^3, $Z = 8$, $\rho_{calcd} = 2.109$ g·cm^{-3}, $\mu = 11.563$ mm^{-1}, $F(000) = 3784$, $T = 100(2)$ K, $R_1 = 0.0240$, $wR^2 = 0.0498$, 6471 independent reflections [2θ≤52.742°] and 301 parameters.

Crystal data for 2-Br: $C_{28}H_{46}BBr_3P_4Pt_2$, $M_r = 1147.25$, colorless block, 0.475×0.315×0.216 mm^3, orthorhombic space group *Pbcn*, $a = 16.7809(6)$ Å, $b = 18.4376(7)$ Å, $c = 11.9317(4)$ Å, $V = 3691.7(2)$ Å^3, $Z = 4$, $\rho_{calcd} = 2.064$ g·cm^{-3}, $\mu = 11.008$ mm^{-1}, $F(000) = 2160$, $T = 100(2)$ K, $R_1 = 0.0223$, $wR^2 = 0.0540$, 3642 independent reflections [2θ≤52.044°] and 179 parameters.

Refinement details for 2-NMe$_2$: Refined as a two-component inversion twin. The structure was refined using the TWIN keyword, the BASF parameter was refined to 0.02. The asymmetric unit contains two toluene molecules, which are each twofold disordered. The first (RESI 3 and 31 TOL) was modelled with a 79:21 ratio disorder, the second (RESI 4 and 5 TOL2) in a 54:46 ratio. In both cases ADPs were restrained with SIMU 0.005 and ISOR 0.005, and the Aratic rings modelled with AFIX 66.

Crystal data for 2-NMe$_2$: $C_{12}H_{34}BBr_2NP_4Pt_2{\cdot}(C_7H_8)_2$, $M_r = 1061.36$, yellow plate, 0.296×0.181×0.103 mm^3, monoclinic space group $P2_1$, $a = 9.9422(3)$ Å, $b = 13.2747(4)$ Å, $c = 14.0023(4)$ Å, b = 105.5020(10)°, $V = 1780.79(9)$ Å^3, $Z = 2$, r$_{calcd}$ = 1.979 g·cm^{-3}, m = 10.287 mm^{-1}, $F(000) = 1008$, $T = 100(2)$ K, $R_1 = 0.0283$, $wR^2 = 0.0506$, 7547 independent reflections [2θ≤53.506°] and 430 parameters.

Crystal data for 3: $C_{10}H_{28}Br_2P_4Pt_2$, $M_r = 822.20$, orange plate, 0.291×0.20×0.129 mm^3, monoclinic space group $C2/c$, $a = 19.3666(10)$ Å, $b = 8.5391(4)$ Å, $c = 14.8224(13)$ Å, $\beta = 123.3850(10)$°, $V = 2046.8(2)$ Å^3, $Z = 4$, $\rho_{calcd} = 2.668$ g·cm^{-3}, $\mu = 17.857$ mm^{-1}, $F(000) = 1496$, $T = 100(2)$ K, $R_1 = 0.0155$, $wR^2 = 0.0379$, 2012 independent reflections [2θ≤52.044°] and 86 parameters.

Crystal data for 4: $C_{14}H_{40}B_2Br_2N_2P_4Pt_2$, $M_r = 931.98$, orange block, 0.18×0.259×0.265 mm^3, Orthorhombic space group $Pbca$, $a = 11.801(7)$ Å, $b = 16.002(13)$ Å, $c = 29.549(10)$ Å, $V = 5580(6)$ Å^3, $Z = 8$, $\rho_{calcd} = 2.219$ g·cm^{-3}, $\mu = 13.115$ mm^{-1}, $F(000) = 3472$, $T = 100(2)$ K, $R_1 = 0.0312$, $wR^2 = 0.0652$, 5664 independent reflections [2θ≤52.736°] and 247 parameters.

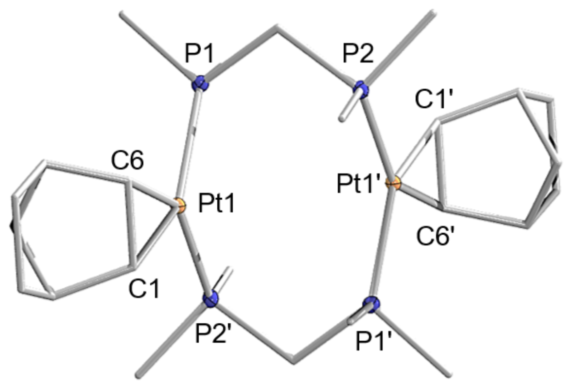

Figure S22. Crystallographically-derived molecular structure of **1**. Thermal ellipsoids drawn at the 50% probability level. Selected carbon ellipsoids and hydrogen atoms have been omitted for clarity. Selected bond lengths (Å) and angles (°): Pt1···Pt2 4.096(1), Pt1−P1 2.2666(8), Pt1−P2 2.268(1), P1−Pt1−P2 107.91(3).

Computational details

Initially, we performed geometry optimizations and Hessian calculations starting from the crystal structures of **2-Y** (Y = Dur, Br, 2-NMe_2) and **4** at the density functional theory (DFT) level using the long-range corrected ωB97XD[6] functional. We applied the adjusted effective core potential (ECP) basis set aug-cc-pVDZ-PP[7] for platinum, while the remaining atoms were described by cc-pVDZ[8-10] basis set. All stationary points were fully characterized as minimum energy structures as no imaginary frequencies were observed.

With the goal of investigating the bonding situation in **2-Y** and **4**, we used the Energy Decomposition Analysis methodology combined with the Natural Orbitals for Chemical Valence (EDA-NOCV).[11] Briefly, the molecule is separated into two distinct fragments, and the interaction energy ΔE_{int} of these fragments is decomposed in three distinct contributions[12–14]:

$$\Delta E_{int} = \Delta E_{elstat} + \Delta E_{Pauli} + \Delta E_{orb} \qquad (S1)$$

where ΔE_{elstat} is the quasi-classical electrostatic attraction, ΔE_{Pauli} is the exchange repulsion and ΔE_{orb} is the orbital interaction energy. The first term is obtained by superimposing the frozen charge densities of the fragments at the optimized geometry of the molecule. The ΔE_{Pauli} term is obtained by antisymmetrizing and renormalizing the product wavefunction. Finally, ΔE_{orb} is obtained by allowing relaxation of the molecular orbitals to their final form. In the EDA-NOCV approach, the ΔE_{orb} term is calculated by considering the Extended Transition State (ETS) method[11,15] expressed in terms of NOCVs. The ψ_i eigenvectors are the ones that diagonalize the deformation density matrix ΔP in the basis of symmetrized fragment orbitals (SFOs):

$$\Delta \mathrm{P}\psi_i = \nu_i \psi_i \qquad (S2)$$

The deformation density $\Delta\rho$ is obtained by the complementary eigenfunctions ψ_{-k} and ψ_k corresponding to eigenvalues ν_{-k} and ν_k in the following manner:

$$\Delta\rho(\mathrm{r}) = \sum \Delta\rho_k(r) = \sum \nu_k[-\psi_{-k}^2(r) + \psi_k^2(r)] \qquad (S3)$$

where k represents the NOCV donor-acceptor pairs. The orbital interaction ΔE_{orb} term is then given by:

$$\Delta E_{orb} = \sum \Delta \mathrm{E}_{k}^{orb} = \sum \nu_k(-F_{-k}^{TS} + F_{k}^{TS}) \qquad (S4)$$

where $-F_{-k}^{TS}$ and F_{k}^{TS} are diagonal transition-state Kohn-Sham matrix elements corresponding to NOCVs with eigenvalues ν_{-k} and ν_k.

The EDA-NOCV analysis was conducted at the B3LYP/TZ2P level of theory.[16-19] Scalar relativistic effects are taken into account by applying the zeroth order regular approximation (ZORA)[20-22] in all EDA-NOCV calculations. The calculations were carried out considering two distinct scenarios: 1) the interaction of the $\{(\mu\text{-dmpm})\mathrm{Pt}\}_2$ and BY fragments in their electronic triplet spin states, corresponding to the formation of electron-sharing Pt–B σ bonds in **2-Y**, and 2) the interaction of both fragments in their electronic singlet states, corresponding to borylene-type complexes with delocalized donor-acceptor bonding.

All geometry optimizations and Hessian calculations were performed with the Gaussian 16, Revision B.01 program.[23] The EDA-NOCV analysis was performed using the ADF 2018 software.[24] Frontier orbitals, images of the 3D structures and deformation densities were visualized and plotted with ADFview.

Table S1. EDA-NOCV results (kcal mol^{-1}) for **2-Dur**.

Energy Term	Triplet Fragments (electron-sharing bonds)	Singlet Fragments (donor-acceptor bonds)
ΔE_{int}	–170.5	–108.5
ΔE_{Pauli}	465.1	783.7
ΔE_{elstat}[*]	–359.3 (56.5%)	–463.3 (51.9%)
ΔE_{orb}[*]	–276.3 (43.5%)	–428.9 (48.1%)
$\Delta E_{orb(1)}$[†]	–131.2 (47.5%)	–352.8 (82.3%)
$\Delta E_{orb(2)}$[†]	–91.7 (33.2%)	–24.6 (5.7%)
$\Delta E_{orb(3)}$[†]	–19.8 (7.2%)	–15.8 (3.7%)
$\Delta E_{orb(4)}$[†]	–7.3 (2.6%)	–10.6 (2.5%)
$\Delta E_{orb(rest)}$[†]	–26.3 (9.5%)	–25.5 (5.8%)

[*]The values in parentheses show the weight of each contribution with respect to the total attractive interaction.

[†]The values in parentheses show the weight of each contribution with respect to the total orbital interaction, ΔE_{orb}.

Table S2. EDA-NOCV results (kcal mol^{-1}) for **2-Br**.

Energy Term	Triplet Fragments (electron-sharing bonds)	Singlet Fragments (donor-acceptor bonds)
ΔE_{int}	–194.4	–117.8
ΔE_{Pauli}	505.2	708.9
ΔE_{elstat}[*]	–433.8 (62.0%)	–388.6 (47.0%)
ΔE_{orb}[*]	–265.8 (38.0%)	–438.1 (53.0%)
$\Delta E_{orb(1)}$[†]	–121.2 (45.6%)	–354.1 (80.8%)
$\Delta E_{orb(2)}$[†]	–88.7 (33.3%)	–30.8 (7.0%)
$\Delta E_{orb(3)}$[†]	–24.5 (9.2%)	–15.3 (3.5%)
$\Delta E_{orb(4)}$[†]	–12.6 (4.7%)	–15.5 (3.5%)
$\Delta E_{orb(rest)}$[†]	–18.9 (7.1%)	–22.4 (5.1%)

[*]The values in parentheses show the weight of each contribution with respect to the total attractive interaction.

[†]The values in parentheses show the weight of each contribution with respect to the total orbital interaction, ΔE_{orb}.

Table S3. EDA-NOCV results (kcal mol^{-1}) for **2-NMe$_2$**.

Energy Term	Triplet Fragments (electron-sharing bonds)	Singlet Fragments (donor-acceptor bonds)
ΔE_{int}	–175.5	–106.4
ΔE_{Pauli}	487.3	714.1
ΔE_{elstat}[*]	–448.9 (67.7%)	–410.6 (50.0%)
ΔE_{orb}[*]	–213.8 (32.3%)	–409.8 (50.0%)
$\Delta E_{orb(1)}$[†]	–89.0 (41.6%)	–350.2 (85.5%)
$\Delta E_{orb(2)}$[†]	–89.6 (41.9%)	–16.3 (4.0%)
$\Delta E_{orb(3)}$[†]	–14.1 (6.6%)	–14.8 (3.6%)
$\Delta E_{orb(4)}$[†]	–6.4 (3.0%)	–11.8 (2.9%)
$\Delta E_{orb(rest)}$[†]	–14.6 (6.8%)	–16.8 (4.1%)

[*]The values in parentheses show the weight of each contribution with respect to the total attractive interaction.

[†]The values in parentheses show the weight of each contribution with respect to the total orbital interaction, ΔE_{orb}.

Table S4. EDA-NOCV results (kcal mol^{-1}) for **4**.

Energy Term	Triplet Fragments (electron-sharing bonds)	Singlet Fragments (donor-acceptor bonds)
ΔE_{int}	–142.5	–160.9
ΔE_{Pauli}	519.9	548.2
ΔE_{elstat}[*]	–440.6 (66.5%)	–445.5 (62.8%)
ΔE_{orb}[*]	–221.9 (33.5%)	–263.6 (37.2%)
$\Delta E_{orb(1)}$[†]	–93.5 (42.1%)	–106.8 (40.5%)
$\Delta E_{orb(2)}$[†]	–86.1 (38.8%)	–112.9 (42.8%)
$\Delta E_{orb(3)}$[†]	–11.4 (5.1%)	–12.2 (4.6%)
$\Delta E_{orb(4)}$[†]	–7.5 (3.4%)	–8.0 (3.0%)
$\Delta E_{orb(rest)}$[†]	–23.4 (10.6%)	–23.7 (9.0%)

[*]The values in parentheses show the weight of each contribution with respect to the total attractive interaction.

[†]The values in parentheses show the weight of each contribution with respect to the total orbital interaction, ΔE_{orb}.

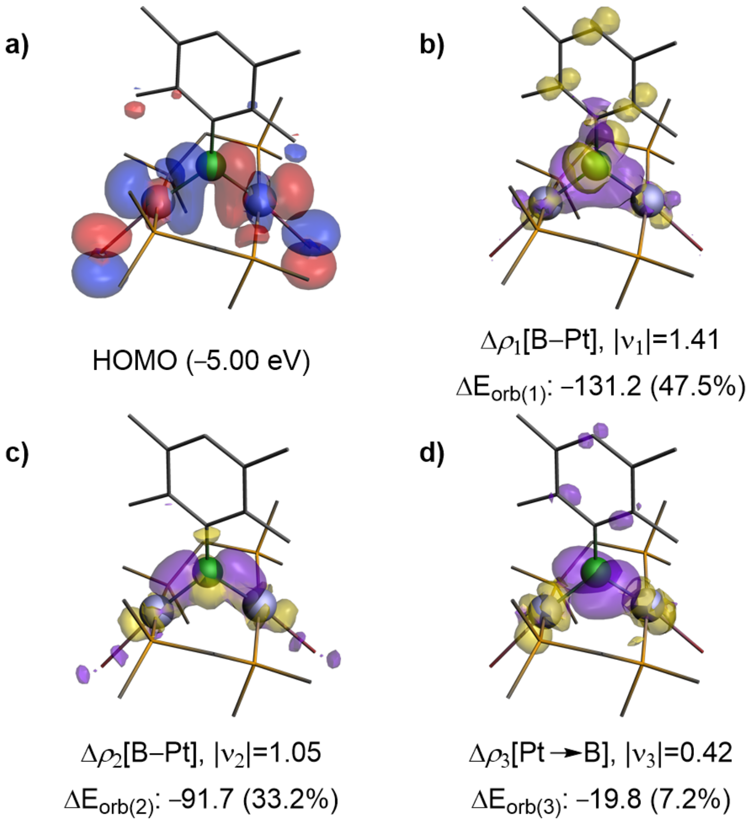

Figure S23. a) Plot of the HOMO of **2-Dur** at the B3LYP/TZV2P//ωB97XD/cc-pVDZ,aug-cc-pVDZ-PP{Pt} level of theory. b-d) Plot of deformation densities ($\Delta\rho_k$), at the same level of theory, of the main orbital interactions of **2-Dur** starting from fragments at their electronic triplet states.

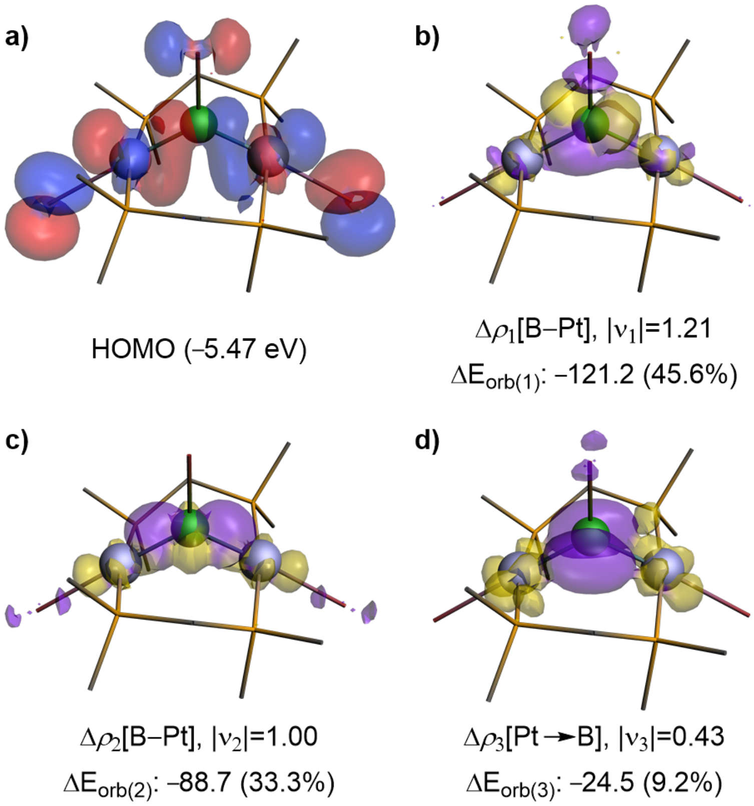

Figure S24. a) Plot of the HOMO of **2-Br** at the B3LYP/TZV2P//ωB97XD/cc-pVDZ,aug-cc-pVDZ-PP{Pt} level of theory. b-d) Plot of deformation densities ($\Delta\rho_k$), at the same level of theory, of the main orbital interactions of **2-Br** starting from fragments at their electronic triplet states.

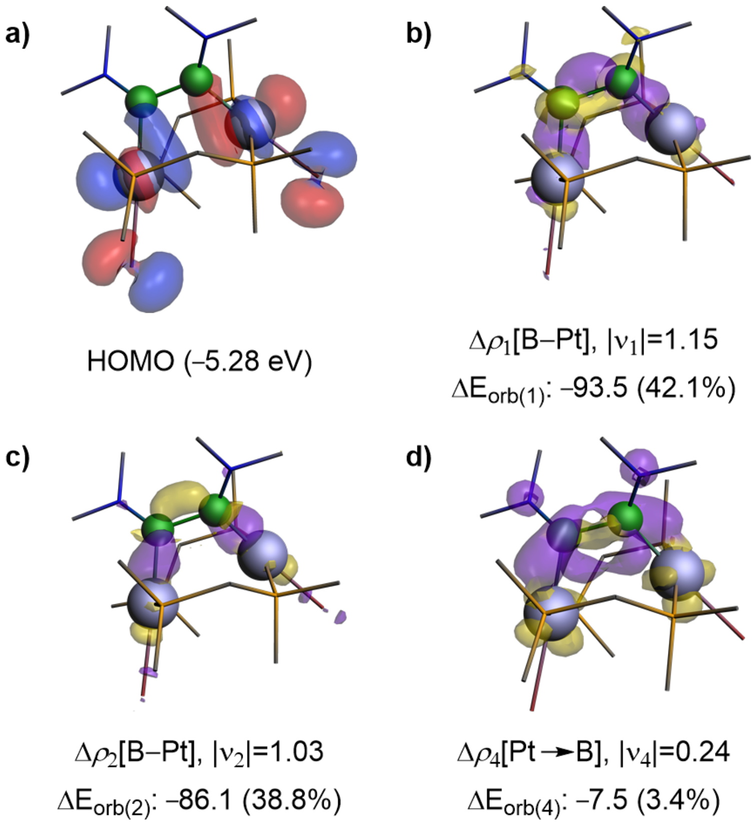

Figure S24. a) Plot of the HOMO of **4** at the B3LYP/TZV2P//ωB97XD/cc-pVDZ,aug-cc-pVDZ-PP{Pt} level of theory. b-d) Plot of deformation densities ($\Delta\rho_k$), at the same level of theory, of the main orbital interactions of **4** starting from fragments at their electronic triplet states.

Cartesian Coordinates and Energies

2-Dur

E(ωB97XD/cc-pVDZ,aug-cc-pVDZ-PP{Pt}) = –7564.704910 E_h

ΔΔG(298.15) = 0.499528 E_h

Lowest frequency = 11.73 cm^{-1}

P	-0.128358000	-1.864095000	2.158986000
C	0.814010000	-2.437216000	-3.458087000
H	1.461989000	-3.240377000	-3.078730000
H	1.132316000	-2.132663000	-4.465988000
H	-0.220741000	-2.806737000	-3.487387000
P	0.920461000	1.048088000	2.273203000
C	-0.159066000	0.241294000	-3.058248000
H	0.097610000	0.380066000	-4.120463000
H	-1.188781000	-0.146390000	-3.000984000
Br	2.340445000	-3.366551000	-0.108686000
Pt	0.413458000	-1.573653000	-0.054294000
C	0.995310000	2.897176000	-3.163163000
H	1.030136000	3.901178000	-2.718381000
H	0.653449000	2.946545000	-4.207604000
H	2.010846000	2.482944000	-3.109605000
P	0.920965000	-1.047205000	-2.273283000
Br	2.336733000	3.369256000	0.108258000
B	-0.890856000	-0.000507000	0.000099000
Pt	0.411803000	1.574031000	0.054318000
P	-0.130744000	1.863828000	-2.158896000
C	-1.787789000	2.564863000	-2.502173000
H	-2.544832000	1.950636000	-1.994047000
H	-1.990755000	2.602799000	-3.582523000
H	-1.822806000	3.581197000	-2.084169000
C	2.621574000	-0.405077000	-2.472048000
H	2.752018000	0.475707000	-1.825291000
H	2.833008000	-0.149211000	-3.521099000
H	3.306520000	-1.191686000	-2.124802000

C	0.998641000	-2.896634000	3.163036000
H	1.034327000	-3.900531000	2.718088000
H	0.656809000	-2.946493000	4.207467000
H	2.013815000	-2.481495000	3.109556000
C	-0.157879000	-0.241647000	3.058426000
H	-1.188005000	0.145013000	3.001635000
H	0.099350000	-0.380224000	4.120533000
C	0.812163000	2.437913000	3.458111000
H	1.131140000	2.133701000	4.465902000
H	-0.223042000	2.806143000	3.487721000
H	1.459029000	3.241902000	3.078610000
C	2.621804000	0.407810000	2.471492000
H	2.833759000	0.152004000	3.520450000
H	3.305890000	1.195157000	2.124219000
H	2.752988000	-0.472732000	1.824562000
C	-1.784798000	-2.566449000	2.502532000
H	-1.819054000	-3.582832000	2.084578000
H	-2.542407000	-1.952864000	1.994480000
H	-1.987581000	-2.604509000	3.582915000
C	-2.493216000	-0.001273000	0.000157000
C	-3.230325000	-1.011056000	-0.691474000
C	-4.637144000	-0.984284000	-0.717473000
C	-5.311405000	-0.002713000	0.000194000
H	-6.405468000	-0.003260000	0.000191000
C	-4.638123000	0.979548000	0.717855000
C	-3.231326000	1.007762000	0.691830000
C	-2.579034000	-2.146146000	-1.445268000
H	-1.544405000	-2.316953000	-1.117175000
H	-2.568004000	-1.954438000	-2.533575000
H	-3.132026000	-3.085371000	-1.304176000
C	-5.434598000	-2.005479000	-1.491169000
H	-5.318091000	-3.018908000	-1.073576000
H	-5.119571000	-2.058239000	-2.544922000
H	-6.505642000	-1.760741000	-1.472300000

C	-5.436624000	1.999910000	1.491577000
H	-6.507432000	1.754149000	1.472582000
H	-5.321039000	3.013501000	1.074127000
H	-5.121729000	2.052839000	2.545364000
C	-2.581173000	2.143517000	1.445619000
H	-2.569521000	1.951608000	2.533881000
H	-3.135369000	3.082082000	1.304898000
H	-1.546883000	2.315755000	1.117222000

2-Br

E(ωB97XD/cc-pVDZ,aug-cc-pVDZ-PP{Pt}) = –9750.116570 E_h

ΔΔG(298.15) = 0.307779 E_h

Lowest frequency = 12.80 cm^{-1}

Pt	0.000000000	-1.718298000	0.017610000
B	0.000000000	0.000000000	1.030718000
Br	0.000000000	0.000000000	3.048605000
Br	-0.069613000	-3.904544000	-1.409428000
P	2.273500000	1.394158000	-0.309667000
P	2.297372000	-1.712694000	0.271752000
C	2.630090000	1.104389000	-2.079926000
H	2.323508000	2.008087000	-2.626149000
H	2.012624000	0.266000000	-2.432786000
H	3.696491000	0.894682000	-2.250882000
C	3.052142000	-0.041864000	0.568803000
H	4.131014000	-0.078650000	0.347481000
H	2.931754000	0.169044000	1.644389000
C	3.363981000	2.782708000	0.164343000
H	3.289675000	2.940586000	1.249457000
H	2.988399000	3.681284000	-0.346056000
H	4.409207000	2.588872000	-0.117682000
C	3.254420000	-2.389057000	-1.132751000
H	4.332419000	-2.379478000	-0.914079000
H	3.047747000	-1.803849000	-2.038048000

H	2.902534000	-3.415010000	-1.310889000
C	2.888539000	-2.674169000	1.711894000
H	3.982093000	-2.615631000	1.814695000
H	2.580657000	-3.719641000	1.567167000
H	2.400172000	-2.286086000	2.616934000
Pt	0.000000000	1.718298000	0.017610000
Br	0.069613000	3.904544000	-1.409428000
P	-2.273500000	-1.394158000	-0.309667000
P	-2.297372000	1.712694000	0.271752000
C	-2.630090000	-1.104389000	-2.079926000
H	-2.323508000	-2.008087000	-2.626149000
H	-2.012624000	-0.266000000	-2.432786000
H	-3.696491000	-0.894682000	-2.250882000
C	-3.052142000	0.041864000	0.568803000
H	-4.131014000	0.078650000	0.347481000
H	-2.931754000	-0.169044000	1.644389000
C	-3.363981000	-2.782708000	0.164343000
H	-3.289675000	-2.940586000	1.249457000
H	-2.988399000	-3.681284000	-0.346056000
H	-4.409207000	-2.588872000	-0.117682000
C	-3.254420000	2.389057000	-1.132751000
H	-4.332419000	2.379478000	-0.914079000
H	-3.047747000	1.803849000	-2.038048000
H	-2.902534000	3.415010000	-1.310889000
C	-2.888539000	2.674169000	1.711894000
H	-3.982093000	2.615631000	1.814695000
H	-2.580657000	3.719641000	1.567167000
H	-2.400172000	2.286086000	2.616934000

2-NMe_2

E(ωB97XD/cc-pVDZ,aug-cc-pVDZ-PP{Pt}) = –7310.463012 E_h

ΔΔG(298.15) = 0.387006 E_h

Lowest frequency = 11.31 cm^{-1}

Pt	1.679626000	-0.126535000	0.092165000
C	2.590293000	3.073747000	-1.039320000
H	2.647521000	4.150237000	-0.819465000
H	1.989912000	2.903420000	-1.941760000
H	3.591385000	2.660553000	-1.227194000
Br	3.758894000	-0.345845000	-1.499774000
P	1.869846000	2.151355000	0.368897000
Pt	-1.679685000	0.126686000	0.092349000
C	2.914220000	2.689032000	1.776941000
H	2.961134000	3.785968000	1.846871000
H	3.924313000	2.282883000	1.622804000
H	2.507386000	2.272826000	2.708215000
Br	-3.759297000	0.346369000	-1.499120000
P	-1.869773000	-2.151358000	0.368027000
C	0.900778000	-2.767165000	-1.942562000
H	0.604667000	-3.818068000	-2.078568000
H	1.830385000	-2.552656000	-2.489213000
H	0.120670000	-2.094465000	-2.326168000
P	-1.209470000	2.369133000	-0.183124000
C	-2.589356000	-3.073000000	-1.041120000
H	-2.645497000	-4.149784000	-0.822432000
H	-1.989269000	-2.901040000	-1.943440000
H	-3.590881000	-2.660608000	-1.228452000
C	-2.914770000	-2.690062000	1.775209000
H	-2.961881000	-3.787051000	1.844178000
H	-3.924735000	-2.283616000	1.621035000
H	-2.508230000	-2.274730000	2.707006000
C	-0.899936000	2.766724000	-1.942367000
H	-0.604211000	3.817705000	-2.078637000
H	-1.829206000	2.551642000	-2.489368000

H	-0.119356000	2.094205000	-2.325348000
C	-2.526414000	3.538274000	0.315102000
H	-2.268036000	4.575422000	0.055101000
H	-2.693111000	3.453900000	1.398324000
H	-3.445495000	3.231457000	-0.205061000
C	-0.272695000	-3.031055000	0.711200000
H	-0.061540000	-2.885572000	1.783244000
H	-0.386710000	-4.110828000	0.523240000
C	0.272738000	3.031166000	0.711679000
H	0.386858000	4.110893000	0.523524000
H	0.061337000	2.885938000	1.783706000
C	2.526647000	-3.537877000	0.315618000
H	2.268788000	-4.575065000	0.055257000
H	2.692558000	-3.453653000	1.398973000
H	3.445984000	-3.230688000	-0.203874000
P	1.209678000	-2.369056000	-0.183332000
B	0.000013000	-0.000143000	1.267382000
N	0.000045000	-0.000709000	2.690125000
C	1.188991000	-0.193683000	3.498378000
H	1.358590000	0.663739000	4.177610000
H	2.066641000	-0.315110000	2.849739000
H	1.094666000	-1.092783000	4.136260000
C	-1.188950000	0.191181000	3.498565000
H	-1.358664000	-0.667216000	4.176544000
H	-2.066541000	0.313592000	2.850034000
H	-1.094605000	1.089347000	4.137745000

4

E(ωB97XD/cc-pVDZ,aug-cc-pVDZ-PP{Pt}) = –7469.879724 E_h

ΔΔG(298.15) = 0.473557 E_h

Lowest frequency = 25.43 cm^{-1}

C	0.602132000	2.937883000	0.547942000
H	0.841984000	3.984650000	0.301035000
H	0.218875000	2.905775000	1.579176000
Pt	1.714871000	-0.263298000	-0.019952000
Br	2.994505000	-0.234391000	-2.322419000
P	2.163538000	1.924416000	0.507606000
C	-0.602344000	-2.950026000	0.478481000
H	-0.218501000	-2.942617000	1.509951000
H	-0.842413000	-3.990564000	0.206752000
Pt	-1.715037000	0.263597000	-0.013060000
P	0.780438000	-2.320973000	-0.591541000
Br	-2.995984000	0.289410000	-2.314827000
C	3.239633000	2.862263000	-0.638319000
H	2.822570000	2.807246000	-1.652229000
H	4.223902000	2.374557000	-0.666995000
H	3.338687000	3.909012000	-0.314020000
P	-2.163630000	-1.935716000	0.463135000
C	2.903349000	2.295337000	2.145626000
H	2.987540000	3.379439000	2.313249000
H	3.902203000	1.837510000	2.186318000
H	2.282807000	1.843111000	2.930746000
P	-0.780934000	2.334251000	-0.536312000
C	0.076935000	-2.364027000	-2.278474000
H	-0.690433000	-1.581482000	-2.380198000
H	-0.354418000	-3.353955000	-2.491424000
H	0.890496000	-2.139128000	-2.981460000
C	1.949426000	-3.733284000	-0.582792000
H	2.330660000	-3.892205000	0.435168000
H	2.791743000	-3.459846000	-1.235036000
H	1.473348000	-4.653966000	-0.951785000

C	-3.240705000	-2.846377000	-0.703598000
H	-4.224637000	-2.357439000	-0.720752000
H	-3.340373000	-3.900200000	-0.403292000
H	-2.823895000	-2.768673000	-1.716114000
C	-2.902183000	-2.344816000	2.092658000
H	-3.900996000	-1.888075000	2.144830000
H	-2.280979000	-1.910969000	2.887575000
H	-2.986185000	-3.432536000	2.235028000
C	-0.077154000	2.416726000	-2.221705000
H	-0.890338000	2.208023000	-2.930070000
H	0.690578000	1.636956000	-2.341115000
H	0.353913000	3.411477000	-2.411486000
C	-1.950212000	3.745734000	-0.493961000
H	-1.474618000	4.674908000	-0.841670000
H	-2.330757000	3.880656000	0.527739000
H	-2.792906000	3.487347000	-1.151843000
B	0.754497000	-0.428419000	1.809065000
C	2.520840000	-1.975675000	2.781857000
H	3.084070000	-1.656335000	1.894948000
H	2.376556000	-3.072989000	2.738688000
H	3.123254000	-1.764453000	3.683604000
N	1.248292000	-1.278853000	2.835604000
B	-0.753313000	0.384914000	1.818884000
N	-1.245849000	1.211437000	2.865388000
C	-0.486954000	1.545019000	4.055268000
H	-0.295235000	2.634482000	4.108699000
H	0.479387000	1.027057000	4.052532000
H	-1.030818000	1.268634000	4.977488000
C	-2.517615000	1.910749000	2.828678000
H	-2.372071000	3.008625000	2.811901000
H	-3.120168000	1.678594000	3.725168000
H	-3.081309000	1.613489000	1.934436000
C	0.490183000	-1.641491000	4.017464000
H	1.033854000	-1.386163000	4.945840000

H	0.300097000	-2.732187000	4.045203000
H	-0.476978000	-1.125117000	4.027225000

References

[1] L. E. Crascall, J. L. Spencer, *Inorg. Synth.* **1990**, *28*, 126–132

[2] H. Braunschweig, Q. Ye, K. Radacki, *Chem. Commun.* **2012**, *48*, 2701–2703.

[3] A. Moezzi, M. M. Olmstead, P. P. Power, *J. Chem. Soc., Dalton Trans.* **1992**, 2429-2434.

[4] G. Sheldrick, *Acta Cryst.*, **2015**, *A71*, 3–8.

[5] G. Sheldrick, *Acta Cryst.*, **2008**, *A64*, 112–122.

[6] J.-D. Chai, M. Head-Gordon, *Phys. Chem. Chem. Phys.* **2008**, *10*, 6615–6620.

[7] D. Figgen, K. A. Peterson, M. Dolg, H. Stoll, *J. Chem. Phys.* **2009**, *130*, 164108.

[8] T. H. Dunning, *J. Chem. Phys.* **1989**, *90*, 1007–1023.

[9] D. E. Woon, T. H. Dunning, *J. Chem. Phys.* **1993**, *98*, 1358–1371.

[10] A. K. Wilson, D. E. Woon, K. A. Peterson, T. H. Dunning, *J. Chem. Phys.* **1999**, *110*, 7667–7676.

[11] M. P. Mitoraj, A. Michalak, T. Ziegler, *J. Chem. Theory Comput.* **2009**, *5*, 962–975.

[12] K. Morokuma, *J. Chem. Phys.* **1971**, *55*, 1236–1244.

[13] T. Ziegler, A. Rauk, *Theor. Chim. Acta* **1977**, *46*, 1–10.

[14] M. von Hopffgarten, G. Frenking, *Wiley Interdiscip. Rev. Comput. Mol. Sci.* **2012**, *2*, 43–62.

[15] M. P. Mitoraj, A. Michalak, T. Ziegler, *Organometallics* **2009**, *28*, 3727–3733.

[16] A. D. Becke, *J. Chem. Phys.* **1993**, *98*, 5648–5652.

[17] C. Lee, W. Yang, R. G. Parr, *Phys. Rev. B* **1988**, *37*, 785–789.

[18] S. H. Vosko, L. Wilk, M. Nusair, *Can. J. Phys.* **1980**, *58*, 1200–1211.

[19] P. J. Stephens, F. J. Devlin, C. F. Chabalowski, M. J. Frisch, *J. Phys. Chem.* **1994**, *98*, 11623–11627.

[20] E. van Lenthe, E. J. Baerends, J. G. Snijders, *J. Chem. Phys.* **1993**, *99*, 4597–4610.

[21] E. van Lenthe, E. J. Baerends, J. G. Snijders, *J. Chem. Phys.* **1994**, *101*, 9783–9792.

[22] E. van Lenthe, A. Ehlers, E.-J. Baerends, *J. Chem. Phys.* **1999**, *110*, 8943–8953.

[23] Gaussian 16, Revision B.01, M. J. Frisch, G. W. Trucks, H. B. Schlegel, G. E. Scuseria, M. A. Robb, J. R. Cheeseman, G. Scalmani, V. Barone, G. A. Petersson, H. Nakatsuji, X. Li, M. Caricato, A. V. Marenich, J. Bloino, B. G. Janesko, R. Gomperts, B. Mennucci, H. P. Hratchian, J. V. Ortiz, A. F. Izmaylov, J. L. Sonnenberg, D. Williams-Young, F. Ding, F. Lipparini, F. Egidi, J. Goings, B. Peng, A. Petrone, T. Henderson, D. Ranasinghe, V. G. Zakrzewski, J. Gao, N. Rega, G. Zheng, W. Liang, M. Hada, M. Ehara, K. Toyota, R. Fukuda, J. Hasegawa, M. Ishida, T. Nakajima, Y. Honda, O. Kitao,

H. Nakai, T. Vreven, K. Throssell, J. A. Montgomery, Jr., J. E. Peralta, F. Ogliaro, M. J. Bearpark, J. J. Heyd, E. N. Brothers, K. N. Kudin, V. N. Staroverov, T. A. Keith, R. Kobayashi, J. Normand, K. Raghavachari, A. P. Rendell, J. C. Burant, S. S. Iyengar, J. Tomasi, M. Cossi, J. M. Millam, M. Klene, C. Adamo, R. Cammi, J. W. Ochterski, R. L. Martin, K. Morokuma, O. Farkas, J. B. Foresman, and D. J. Fox, Gaussian, Inc., Wallingford CT, **2016**.

[24] G. te Velde, F. M. Bickelhaupt, E. J. Baerends, C. Fonseca Guerra, S. J. A. van Gisbergen, J. G. Snijders, T. Ziegler, *J. Comput. Chem.* **2001**, *22*, 931–967.
